# Transcranial direct current stimulation over the primary motor cortex improves speech production in post-stroke dysarthric speakers: A randomized pilot study

**DOI:** 10.1371/journal.pone.0275779

**Published:** 2022-10-13

**Authors:** Min Ney Wong, Faisal Nouman Baig, Yeuk Ki Chan, Manwa L. Ng, Frank F. Zhu, Joseph Shiu Kwong Kwan

**Affiliations:** 1 Department of Chinese and Bilingual Studies, The Hong Kong Polytechnic University, Hung Hom, Kowloon, Hong Kong; 2 Research Institute for Smart Ageing, The Hong Kong Polytechnic University, Hung Hom, Kowloon, Hong Kong; 3 Research Centre for Language, Cognition, and Neuroscience, The Hong Kong Polytechnic University, Hung Hom, Kowloon, Hong Kong; 4 Speech Science Laboratory, Faculty of Education, The University of Hong Kong, Pokfulam, Hong Kong; 5 HealthyMind Neuro-Wellness Centre, Central, Hong Kong; 6 Department of Brain Sciences, Faculty of Medicine, Imperial College London, London, United Kingdom; University of Caytania, ITALY

## Abstract

**Purpose:**

The current study investigated the therapeutic potential of transcranial direct current stimulation (tDCS) on speech intelligibility, speech-related physiological and vocal functions among post-stroke dysarthric patients.

**Method:**

Nine chronic post-stroke dysarthric patients were randomly assigned to the stimulation or sham group. The stimulation group received 2mA of anodal tDCS over the left inferior primary motor cortex for 15 minutes, while the sham group received 30s of stimulation under the same settings. All the participants received 10 daily 15 minutes of individualized speech therapy targeting their dominant phonological process or phonemes with the greatest difficulty. The outcome measures included (1) perceptual analysis of single words, passage reading and diadochokinetic rate, (2) acoustic analysis of a sustained vowel, and (3) kinematic analysis of rapid syllable repetitions and syllable production in sentence, conducted before and after the treatment.

**Results:**

The results revealed that both the stimulation and sham groups had improved perceptual speech intelligibility at the word level, reduced short rushes of speech during passage reading, improved rate during alternating motion rate, AMR-k^h^a1, and improved articulatory kinematics in AMR-t^h^a1 and syllables /t^h^a1/ and /k^h^a1/ production in sentence. Compared to the sham group, the stimulation group showed significant improvement in articulatory kinematics in AMR-k^h^a1 and syllable /k^h^a1/ production in sentence. The findings also showed that anodal stimulation led to reduced shimmer value in sustained vowel /a/ phonation, positive changes in articulatory kinematics in AMR-t^h^a1 and syllables /p^h^a1/ and /k^h^a1/ production in sentence at the post treatment measure. In addition to positive effects on articulatory control, reduced perturbation of voice amplitude documented in the stimulation group post treatment suggests possible tDCS effects on the vocal function.

**Conclusions:**

The current study documented the beneficial effects of anodal tDCS over the primary motor cortex on speech production and suggested that combined tDCS and speech therapy may promote recovery from post-stroke dysarthria.

## Introduction

Dysarthria is an acquired speech disorder affecting speech motor control and execution, resulting from neurological injury to the motor component of speech area and affecting 38–41% of the stroke population [1, 2]. Similar to apraxia of speech (another speech disorder affecting speech motor planning and programming) [3] and aphasia (a language disorder resulting in difficulties using linguistic symbols while listening, speaking, reading and writing) [4], dysarthria is a neurological deficit root in perilesional and diaschisis regions of the brain and have a detrimental influence on one’s ability to communicate and quality of life (QoL). Post-stroke dysarthria can be classified based on the deficits and the site of disruption of the brain area. For example, flaccid dysarthria results from lower motor neuron lesion, spastic dysarthria involves bilateral upper motor neuron lesion, ataxic dysarthria engages the cerebellar control circuit, hypokinetic and hyperkinetic dysarthrias result after damage to the basal ganglia control circuits [5]. The mainstay of dysarthria remains to be individualized speech therapy. It may involve oral motor exercises (such as strengthening and stretching) and articulation exercises (e.g., minimal contrast). Speech therapy targets enhancement of the speech subsystem with a focus on behavioral changes (e.g., slowing speech rate), kinematic and somatosensory aspects for improvement of speech intelligibility. However, speech therapy outcomes are relative and time-consuming [6]. There is an urge for robust techniques or therapies that directly potentiate brain cortices to enhance neuroplasticity and rehabilitation after stroke.

Non-invasive brain stimulation techniques, such as transcranial magnetic stimulation (TMS) and transcranial direct current stimulation (tDCS), have acquired overwhelming attention for their tendency to bring brain modulatory effects. TMS causes a brief electric pulse on the brain tissue via a fluctuating magnetic field created by the TMS coil, while tDCS reversibly polarizes brain regions by applying mild direct currents topically [7]. A randomized controlled trial investigated the therapeutic potential of rTMS for recovery from stroke-induced dysarthria showed that although patients in both the real rTMS and sham rTMS group showed improvement in dysarthria scales, the real rTMS group showed greater improvement in the sequential motion rate task when compared to the sham rTMS group. The authors concluded that rTMS can have a synergistic effect with speech therapy in treating dysarthria after stroke [8]. A recent review on therapeutic applications of rTMS in stroke rehabilitation compared the potential of low frequency and high frequency rTMS and found that both the high frequency-and low frequency-rTMS have been shown to be safe and well-tolerated. However, positive effects were not observed for the relief of cognitive impairment and spasticity [9]. The potential role of another type of non-invasive brain stimulation technique, repetitive transorbital alternating current stimulation (rtACS), on stroke recovery was investigated and the results showed that rtACS could modulate brain plasticity and recovery from deficits caused by stroke [10].

In comparison to TMS and other types of non-invasive brain stimulation, tDCS is relatively popular due to its safety profile, portability, and cost-effectiveness [11]. Also, tDCS is a user-friendly technique that offers painless cortical excitability without significant side effects [12]. During delivery of tDCS, a weak direct current of 1–2 mA is delivered via electrodes, with at least one positioned on the scalp while the other placed at a cephalic or extracephalic location such as the shoulder for grounding [13]. Direct current travels from the scalp to the anode’s cortical and subcortical regions and leaves the brain at the cathode. The weak current alters the resting membrane potential to cause depolarization. Consequently, cortical excitation due to the altered firing rate of neuronal impulses induces neuroplasticity [14]. The effects of tDCS depend on current density, duration, site of stimulation, and direction of polarity. Current density is the current intensity over the area of electrode montage. A current density of 0.03 mA/cm^2^ was adequate in inducing an excitability shift in the human primary motor cortex (M1) [15]. The current density that can successfully induce effective stimulation in most studies ranges from 0.029 to 0.08 mA/cm^2^ [16]. Polarity is also a crucial element in determining the effect of tDCS. Current flows inwardly into the cortex from the anode, causing subthreshold depolarization in cortical pyramidal neurons and thus increasing cortical excitability. On the contrary, outward current flow occurs at the cathode causing subthreshold hyperpolarisation in cortical pyramidal neurons and thus decreasing cortical excitability [17].

Efficacy of tDCS for the enhancement of regional cerebral blood flow [18, 19], neuroplasticity and motor rehabilitation [20], treatment of dysarthria [21, 22] and other speech and language disorders [23, 24] has been substantiated through neuroimaging techniques [25, 26]. There is a growing body of evidence in the literature for the usefulness of tDCS for stroke rehabilitation. Two controlled, cross-over design studies investigated the therapeutic potential of tDCS on motor recovery [7, 27], and they consistently demonstrated improved fine motor hand function among post-stroke hemiparesis patients following tDCS application over the primary motor cortex of the affected hemisphere compared to sham groups. Also, the improved behavioral outcomes were associated with increased cortical excitability in the anodal tDCS group only. These changes were demonstrated by the increased recruitment curves and functional magnetic resonance imaging, respectively [7, 28]. Another study [27] further reported that cathodal stimulation in inhibiting the unaffected hemisphere could also improve motor function, similar to the anodal stimulation.

Previous studies have also reported tDCS applications for aphasia and apraxia of speech (AOS) commonly seen in the post-stroke population. Anodal tDCS was reported to enhance therapeutic outcomes in identifying syntactic violation and acquisition of novel lexicon compared to the sham group among post-stroke aphasic patients [29, 30]. Improvements were shown in previously declined cognitive function among post-stroke patients following tDCS sessions across different studies [31, 32]. Two cross-over blinded studies reported the beneficial effect of tDCS on AOS [33, 34]. In both studies, the anodal tDCS electrodes were positioned over the Broca’s area whilst the cathode was placed over the contralateral supraorbital region and the contralesionally homologue of the Broca’s area. Speech therapy intervention for AOS was delivered concurrently, and the entire intervention lasted for 5 and 10 consecutive daily sessions, respectively. The results indicated that articulation accuracy and speed after real stimulation were significantly increased compared to sham condition. The authors further claimed sustained after-effects for 1-week, 1-month and 2-month post intervention. Therefore, it was suggested that anodal tDCS was a promising technique to enhance cortical excitability in the Broca’s area and speech performance in patients with AOS.

Despite the number of studies, the scope of research on tDCS in motor speech disorders is still limited, especially for post-stroke dysarthria. A very recent systemic review on therapeutic potential of non-invasive brain stimulation techniques for the recovery of neurogenic dysarthria included 10 studies with or without concurrent speech therapy in the review. Most studies included in the review reported one or more positive effects of stimulation. However, a concrete conclusion about their efficacy was not established due to high risk of bias and heterogeneity among the included studies [35]. To the authors’ knowledge, only one study had examined the use of tDCS in post-stroke dysarthric patients [21]. The study adopted a randomized double-blinded design and randomly assigned twelve acute stroke dysarthric patients into a stimulation group (anodal tDCS supplemented with conventional speech therapy) and a sham group (conventional speech therapy only). There was a total of 10 treatment sessions over two consecutive weeks. Improvement in maximum phonation time (MPT), alternate motion rate (AMR) and sequential motion rate (SMR) were reported in the stimulation group, while improvements in MPT and SMR were found in the sham group. Between-group comparisons revealed that only AMR-Pa was significantly improved in the stimulation group compared to the sham group. It was suggested that tDCS might serve as an adjuvant strategy to enhance the effect of conventional speech therapy. Although beneficial effects of anodal tDCS in improving dysarthria were reported, the observed improvement may have been contributed by spontaneous recovery. Additionally, the performance in AMR-Pa, a rapid syllable repetition task, may not be generalizable to speech production. Therefore, there is a need to investigate further the effect of tDCS on speech rehabilitation in dysarthria post-stroke, examining a more comprehensive range of tasks using different measures to provide meaningful insight into its effects.

The present study aimed to examine whether conventional tDCS can enhance the effects of traditional speech therapy on speech intelligibility, speech-related physiological functions and vocal function in chronic post-stroke dysarthric patients. Seeing the enhancement effects of tDCS on AOS and dysarthria in previous research, it is hypothesized that anodal stimulation should enhance speech intelligibility compared to sham stimulation. However, based on the difference in nature of AOS and dysarthria (which are the disorder of motor planning/programming and control/execution, respectively), the anode in the present study was placed over the left inferior primary motor cortex (C5), which is similar to You et al [21] but different to some other previous studies where the stimulation site was Broca’s area [33, 34]. This modification was made because Broca’s area is responsible for programming and sending articulatory code to the motor cortex instead of implementation [36]. Therefore, it is hypothesized that placing an anode over the major motor execution centre, the primary motor cortex, would induce more beneficial effects on dysarthria recovery.

## Methods

The current study was a randomized controlled trial. The study was approved by the Institutional Review Board of the University of Hong Kong/Hospital Authority Hong Kong West Cluster, Hong Kong (reference number UW 16–126) and was registered at the ClinicalTrials.gov (study identifier: NCT05497362) and HKU Clinical Trial Registry (study identifier: HKUCTR-2017). All procedures were performed according to relevant guidelines and recommendations of the World Medical Association Declaration of Helsinki: Ethical principles for medical research involving human subjects, 2008. The study was unable to secure funding for its full implementation. Hence the protocol was simplified and only a small sample was collected to serve as a pilot study. The study was changed from a double-blinded design to a single-blinded design, used of conventional tDCS instead of high-definition tDCS, and reduced two secondary outcome measures including strength and endurance measures as well as quality of life measure.

### Participants

**Fig 1:**
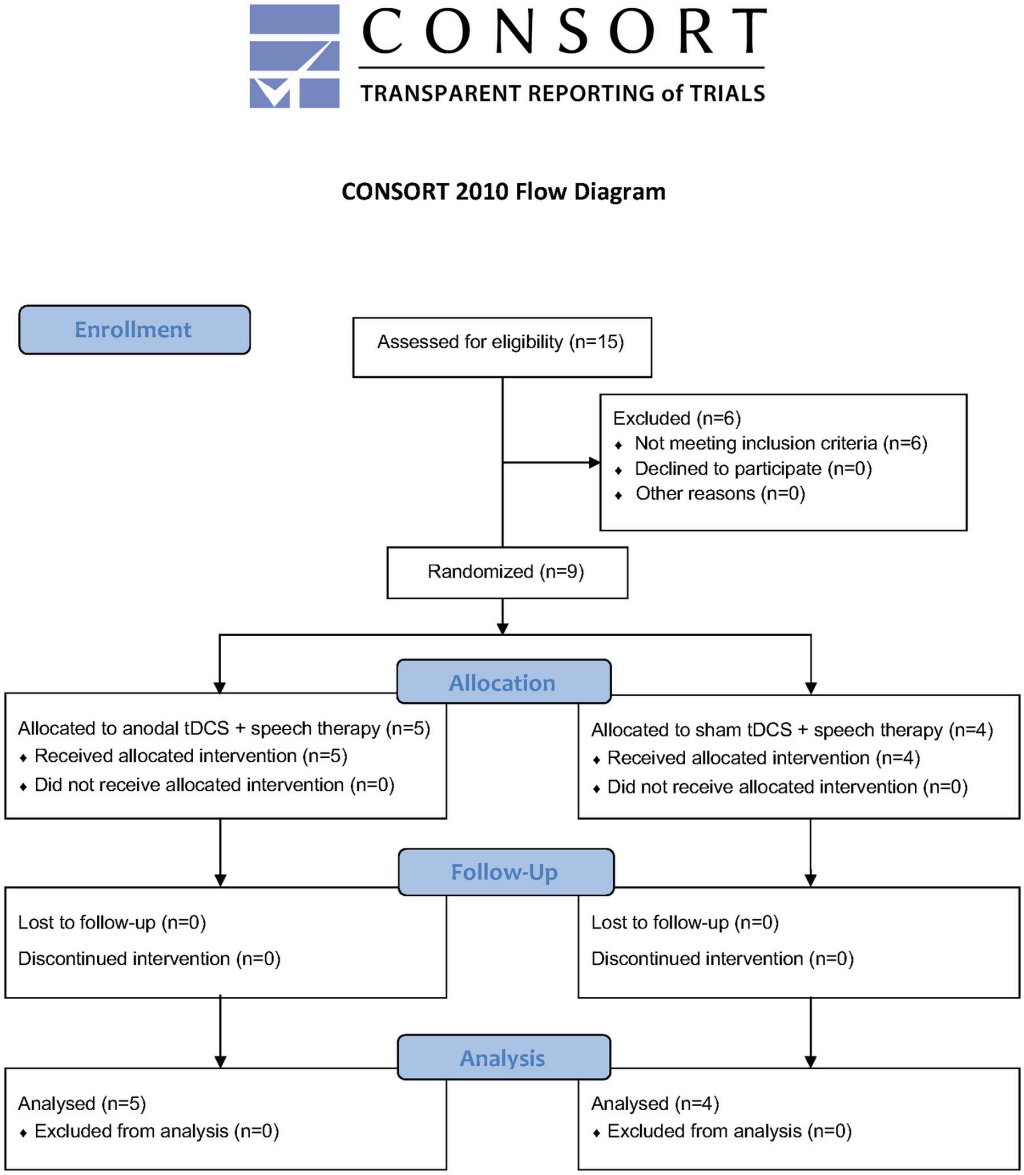
The CONSORT 2010 flow diagram showing the progress through the phases of this study.

Nine post-stroke dysarthric patients (five men and four women) with a mean age of 59.2 ± 6.8 years were recruited from a local hospital and the community stroke support groups. Well-informed written consents were obtained from the participants before the commencement of this study. All participants were native Cantonese speakers with chronic (two to seven years; mean = 4.2 years), unilateral, left hemispheric, single stroke lesions. Individuals with a personal or family history of epilepsy or seizures; a history of a neurological condition, speech disorders, oro-maxillo-facial surgery involving the tongue and lip, severe cognitive impairment, severe aphasia, in a severe or unstable medical condition including heart disease, metallic foreign body implant and any medications that lower neural thresholds (e.g., tricycles, antidepressants, neuroleptic agents, etc.) were excluded from the study. All participants completed previous speech therapy sessions at least six months before the start of this study. Cognitive screening was performed using the Montreal Cognitive Assessment Hong Kong version [37]. Assessment for dysarthria was performed using the Frenchay Dysarthria Assessment- Second Edition (FDA-2) [38] by an experienced, qualified speech therapist before the commencement of the experiments. Screening for aphasia and AOS were also conducted. Four participants had mild aphasia (two in each group), while participants with AOS were excluded from the study. The participants were randomly assigned by a research assistant either to the stimulation (n = 5) or the sham group (n = 4) using a sealed opaque envelope system prepared by the research assistant. When one group is filled, the rest of the participants were assigned to the other group. The participants were blinded about their allocations. All participants completed the treatment and outcome measures. The CONSORT 2010 flow diagram showing the progress through different phases of this study is presented in Fig 1. Table 1 summarizes all participants’ demographic and clinical data in this study.

**Table 1:** Demographic and clinical data of the nine participants.

Participants	Group	Gender	Age	Year post-onset	Severity of dysarthria	Year after the last speech therapy	Educational level
			(years)				
1	Stimulation	M	48	3.5	Mild	3	S
2	Stimulation	M	55	2	Mild	N/A	S
3	Stimulation	F	57	3	Mild	0.5	S
4	Stimulation	M	56	2	Moderate	1.5	S
5	Stimulation	F	61	6	Severe	2	S
6	Sham	F	70	5	Moderate	1	P
7	Sham	F	57	7	Severe	0.5	S
8	Sham	M	69	6	Mild	1	S
9	Sham	M	60	3.5	Mild	1	S

Note: N/A = Not applicable, no previous speech therapy was reported; P = primary school; S = secondary school

### Procedure

Data collection was conducted in a quiet room at the Speech Science Laboratory within the University of Hong Kong over an eight-month period. Pre-treatment baseline data were obtained within one week before treatment. All participants participated in ten consecutive treatment sessions (five days per week, two consecutive weeks). Both stimulation and sham groups received 15 minutes of speech therapy in each treatment session. Concurrently, the stimulation group received 15 minutes of 2mA anodal tDCS, while the sham group only received 30 seconds of stimulation. Post-treatment performance was evaluated and compared with the pre-treatment baseline on the day following the tenth session.

#### Delivery of tDCS

**Fig 2:**
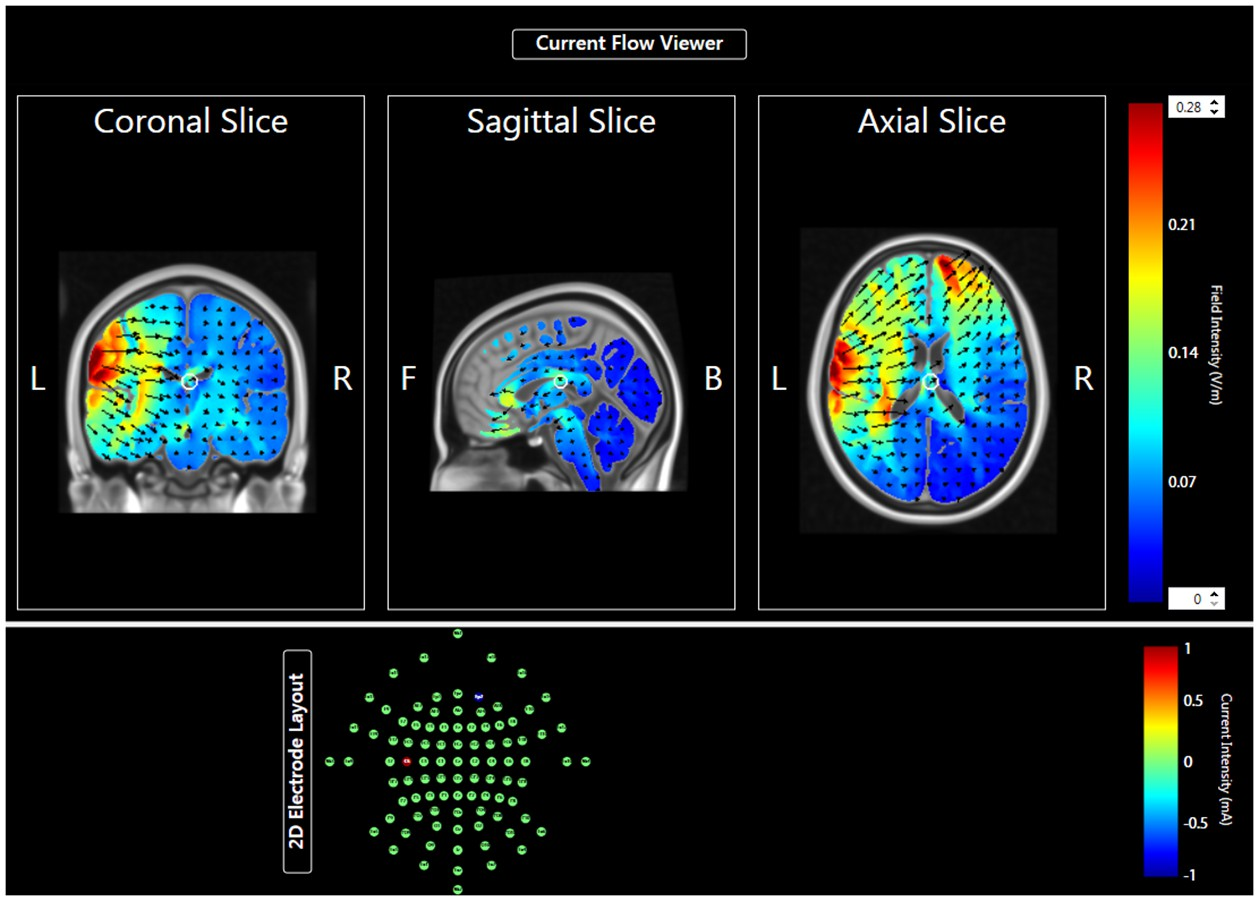
Computer modeling of the current distribution for C5-FP2 montage used in the current study.

tDCS stimulation was delivered using a constant direct current stimulator (Model CID2C, Chattanooga Dual-Channel Iontophoresis Device, Caputron, New York City, USA) with a pair of saline-soaked sponge electrodes (50 x 70 mm) (EasyPads, Soterix Medical Inc., New York, USA). The anodal electrode was placed over the left inferior primary motor cortex (C5) based on the extended International 10–20 System for EEG electrode placement, and the cathodal electrode was positioned on the contralateral supraorbital region. A computer modeling of the current distribution for C5-FP2 montage used in the current study is illustrated in Fig 2. When 2mA direct current was delivered, it produced a current density of 0.057 mA/cm^2^, which was reported to show successful cortical excitability modulation [16]. Such protocol was considered safe as no evidence of tissue damage, heating, or neuronal hyperactivity was reported [39]. The same setting was applied for the sham group, except the stimulator was turned off after 30 seconds to ensure the participants received a slight itching sensation as the stimulation group for blinding purposes.

#### Speech therapy

Individualized speech therapy was provided to all the participants to induce optimal therapy outcomes. Tailor-made speech therapy targeted the participant’s dominant phonological process or phonemes with the greatest difficulty, as identified in the baseline assessment using the Hong Kong Cantonese Articulation Test (HKCAT) [40]. Treatment approaches included minimal pair contrast and phonetic placement, such as providing tactile and visual cues. The participants did not receive additional speech therapy during their participation in the current study.

#### Outcome measures

A series of subjective perceptual evaluation and objective acoustic and kinematic analyses were conducted to examine the participants’ performance on different speech parameters before and after treatment. Table 2 summarises variable outcomes investigated in this study. All the speech production was recorded using a microphone (SM58, Shure, Evanston, IL), a pre-amplification unit (MOTU MicroBook II, Cambridge, MA) and *Praat*. A sampling frequency of 44 kHz and 16 bits/sample quantization rate were used. The microphone was placed approximately 10 cm away from the participant’s mouth. Recordings were done in a soundproof booth to minimize the signal-to-noise ratio.

**Table 2:** Summary of outcome measures investigated in the stimulation and sham groups before and after treatment.

Analysis	Perceptual				Acoustic	Kinematic	
Task	HKCAT	Passage Reading	Diadochokinetic Tasks		Sustained vowel	AMR	Syllable production in sentence
			*AMR*	*SMR*		/p^h^a1/, /t^h^a1/, /k^h^a1/	/p^h^a1/, /t^h^a1/, /k^h^a1/
			/p^h^a1/, /t^h^a1/, /k^h^a1/	/p^h^a1t^h^a1k^h^a1/			
**Parameter measured**	1. Percentage of syllable correct (PSC)	1. Imprecise articulation	1. Rate		1. Maximum phonation time (MPT)	*Approach phase*	
		2. Irregular articulatory breakdown				1. Duration	
						2. Distance	
	2. Percentage of initial consonant correct (PICC)	3. Vowel distortion			2. Jitter	3. Maximum velocity	
		4. Tone distortion			3. Shimmer	4. Maximum acceleration	
		5. Hypernasality			4. Noise-to-harmonics ratio (NHR)	5. Maximum deceleration	
		6. Hyponasality				*Release phase*	
	3. Percentage of vowel or diphthong correct (PVC)	7. Short rushes of speech			5. Harmonic-to-noise ratio (HNR)	6. Duration	
		8. Prolonged intervals				7. Distance	
		9. Prolonged phonemes				8. Maximum velocity	
		10. Repeated phonemes				9. Maximum acceleration	
						10. Maximum deceleration	
	4. Percentage of final consonant correct (PFCC)	11. Rough voice					
		12. Breathy voice					
		13. Strained-strangled voice					
		14. Monoloudness,					
		15. Monopitch					
		16. Excess loudness variation					
		17. Excess pitch variation					
		18. Excess stress					
		19. Overall speech intelligibility					

Note: HKCAT = Hong Kong Cantonese Articulation Test, AMR = alternate motion rate, SMR = sequential motion rate

*Perceptual evaluation*. Perceptual judgments of speech production are widely used in clinical diagnosis and for determining the severity of motor speech disorder [41]. The present study conducted perceptual judgments at the word and passage levels as well as on DDK rate. At the word level, the participants were asked to name 41 pictures from the standardized HKCAT [40] spontaneously. The recorded production was transcribed by two experienced speech therapists to establish inter-rater reliability. Model answers were provided when the participants encountered naming difficulties. The percentage of correctly produced initial consonant (PICC), vowel or diphthong (PVC), and final consonant (PFCC) were calculated. The percentage of the correctly produced syllable (PSC) was then calculated by averaging the transcriptions provided by the two raters.

Passage reading task was included to examine speech characteristics beyond articulatory accuracy measured at the single word. The participants were asked to read aloud a 149-words oral Cantonese passage, “Northwind and the Sun” (see S2 File), a familiar story in Hong Kong. The participants were instructed to read aloud the passage at their regular speaking rate and loudness. Reading by repetition was accepted when the participants demonstrated reading difficulty. The participants’ pre-and post-treatment recordings were then randomized, independently reviewed, and rated by ten final year speech therapy students trained in perceptual analysis of dysarthric speech. The procedures and speech parameters selected were adopted from Whitehill et al [42]. Nineteen speech parameters (see Table 2), including imprecise articulation, irregular articulatory breakdown, vowel distortion, tone distortion, hypernasality, hyponasality, short rushes of speech, prolonged intervals, prolonged phonemes, repeated phonemes, rough voice, breathy voice, strained-strangled voice, monoloudness, monopitch, excess loudness variation, excess pitch variation, excess stress and overall speech intelligibility were rated using a seven-point equal-appearing Likert scale, with a “1” indicating within typical limit performance and a “7” severely deviated from the normal. The ratings by all ten raters were averaged for statistical analysis.

DDK rate is a sensitive measure for articulatory deficits in dysarthric patients [43]. It is often used to assess the speed and regularity of rapid, repetitive articulatory movement, assess articulatory precision, adequacy of velopharyngeal closure, and respiratory and phonatory support [3]. Repetition of single syllable real Cantonese words 趴 /p^h^a1/ (lie on the stomach), 他 /t^h^a1/ (he) and 卡 /k^h^a1/ ([train] car), produced at high-level tone one, were included for AMR, and repetition of /p^h^a1t^h^a1k^h^a1/ was included for SMR. The participants were instructed to repeat the syllables as rapidly as possible in one breath. The DDK rates were calculated by having the number of syllables produced divided by the duration of the production in seconds to obtain the average number of syllables produced per second. The participants repeated each DDK task three times, and their performance across the three repetitions was averaged for further analysis.

*Acoustic analysis*. The target primary motor cortex area covers the control centre for oral articulators and the laryngeal area, crucial in controlling voluntary laryngeal behaviors such as phonation [44]. Therefore, acoustic analyses on sustained vowels were conducted to examine the potential effect of tDCS on the respiratory-phonatory subsystems. The participants were instructed to take a deep breath and sustain the vowel /a/ until they ran out of breath, and the task was repeated five-time. The three most extended productions were selected and averaged for the MPT measure. Acoustic analysis of MPT can quantify pitch, loudness, and voice quality, indicating neurological impairment in the respiratory-phonatory subsystems. Jitter is the variability of fundamental frequency (F0) from one cycle to the next, while shimmer is the amplitude variability from one cycle to the next [45]. Jitter and shimmer are affected by the control of laryngeal muscle over the vocal fold and are correlated with pathologic voice, especially in hoarseness and breathiness [46]. HNR is the ratio between periodic and non-periodic components of a speech sample, while NHR is the ratio between non-periodic and periodic components. Both are used to show speech efficiency. In dysarthric patients, reduced airflow and poor control of vocal folds would increase the noise component in their speech and thus lower their HNR and increase their NHR [47]. Acoustic analysis was performed using the signal analysis software *Praat*.

*Kinematic analysis*. Kinematic data in the present study was collected using the three-dimensional electromagnetic articulography (EMA) AG500 system (Carstens Medizinelektronik GmbH, Bovenden, Germany). It is a non-invasive instrument for tracking articulatory trajectory and computing quantitative kinematic data such as velocity and acceleration [48, 49]. Tongue movement that cannot be directly observed could be examined using the EMA. The participants were seated in a straight-back chair for sensor attachment. The reference sensors were positioned over the left and right mastoid bones and the nasal bridge. The target sensors were attached to the upper lip and lower lip, 1 cm from the tongue tip and tongue dorsum (4 cm from the tongue tip). The stimuli consisted of a rapid repetition of syllables (AMR) 趴 /p^h^a1/, 他 /t^h^a1/ and 卡 /k^h^a1/ and three sentences that have embedded the syllables 趴 /p^h^a1/, 他 /t^h^a1/, and 卡 /k^h^a1/, respectively (see S1 Table). Each target stimulus was produced five times in a randomized manner. If the participant experienced difficulties reading the stimuli presented in written format, they were allowed to repeat after the examiner. Some previous studies have suggested the dissociation between performance in DDK tasks and perceptual speech impairment [50–52]. The inclusion of meaningful sentence stimuli may provide insights into task effects.

**Fig 3:**
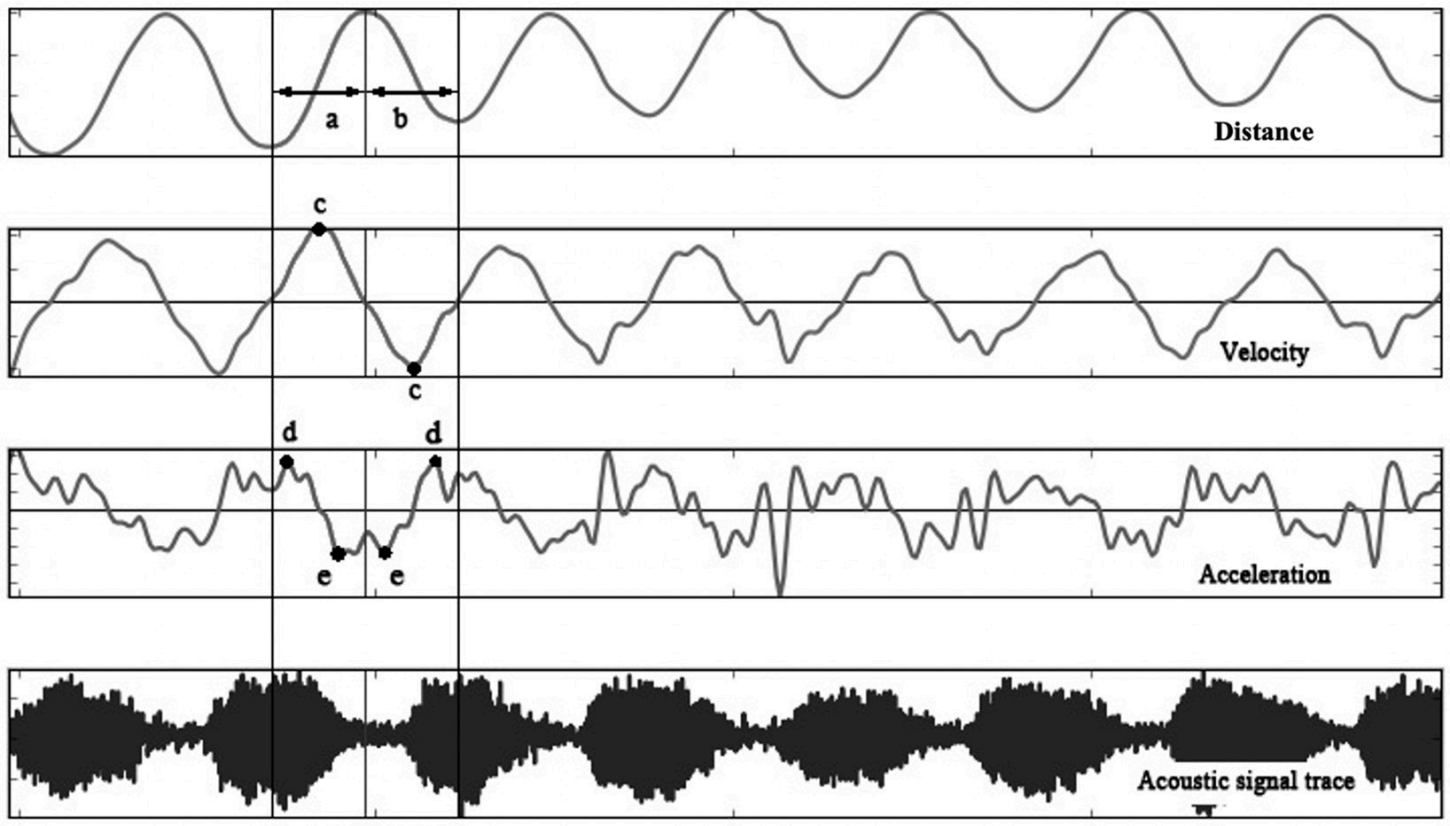
An illustration of kinematic analysis of DDK production using a custom-written EMA analysis programme, Articuno. Note: Example displacement, velocity, acceleration and acoustic signal profiles displaying (a) approach phase, (b) release phase, (c) points of maximum velocity, (d) points of maximum acceleration, and (e) points of maximum deceleration.

A custom-written EMA analysis programme, *Articuno*, was used to annotate and calculate the kinematic measures, including duration (ms), distance (mm), maximum velocity (mm/s), maximum acceleration (m/s^2^) and maximum deceleration (m/s^2^) in the approach (movement towards the upper lip/palate) and release (movement away from the upper lip/palate) phases along the z-axis, i.e., along the mid-sagittal plane. Data from the sensors attached to the lower lip, tongue tip and tongue dorsum were analyzed. An example of the kinematic profile is illustrated in Fig 3.

### Statistical analysis

Data analysis was performed with the Statistical Package for Social Sciences, SPSS (SPSS) software (Version 25, IBM Corporation, Armonk, NY, USA). A series of repeated measure analyses of variance (ANOVA) was performed with *time* as a within-subject factor (pre-tDCS and post-tDCS) and the *group* as a between-subject factor (stimulation and sham groups). Post-hoc pairwise comparisons with Bonferroni adjustment were performed when needed. A *p*-value of 0.05 was adopted as the level of significance. The effect size (Cohen’s *d*) was calculated for parameters that showed significant between group differences and/or significant interaction. The value of Cohen’s *d* is interpreted as the following: small, *d* ≥ 0.2 and <0.5; medium, *d* ≥ 0.5 and <0.8; large, *d* ≥ 0.8 [53, 54]. Cohen’s Kappa Coefficient was used to calculate inter- and intra-rater reliabilities. The value of K is interpreted as the following: weak (0.40–0.59), moderate (0.60–0.79), strong (0.80–0.90) and almost perfect (above 0.90) [55]. Due to small sample size, no subgroup analysis was performed.

## Results

All participants tolerated tDCS well with no adverse effects during or after the treatment. All participants completed the treatment, and none could confirm whether they were in the stimulation or sham group.

### Perceptual evaluation

#### Word level

**Fig 4:**
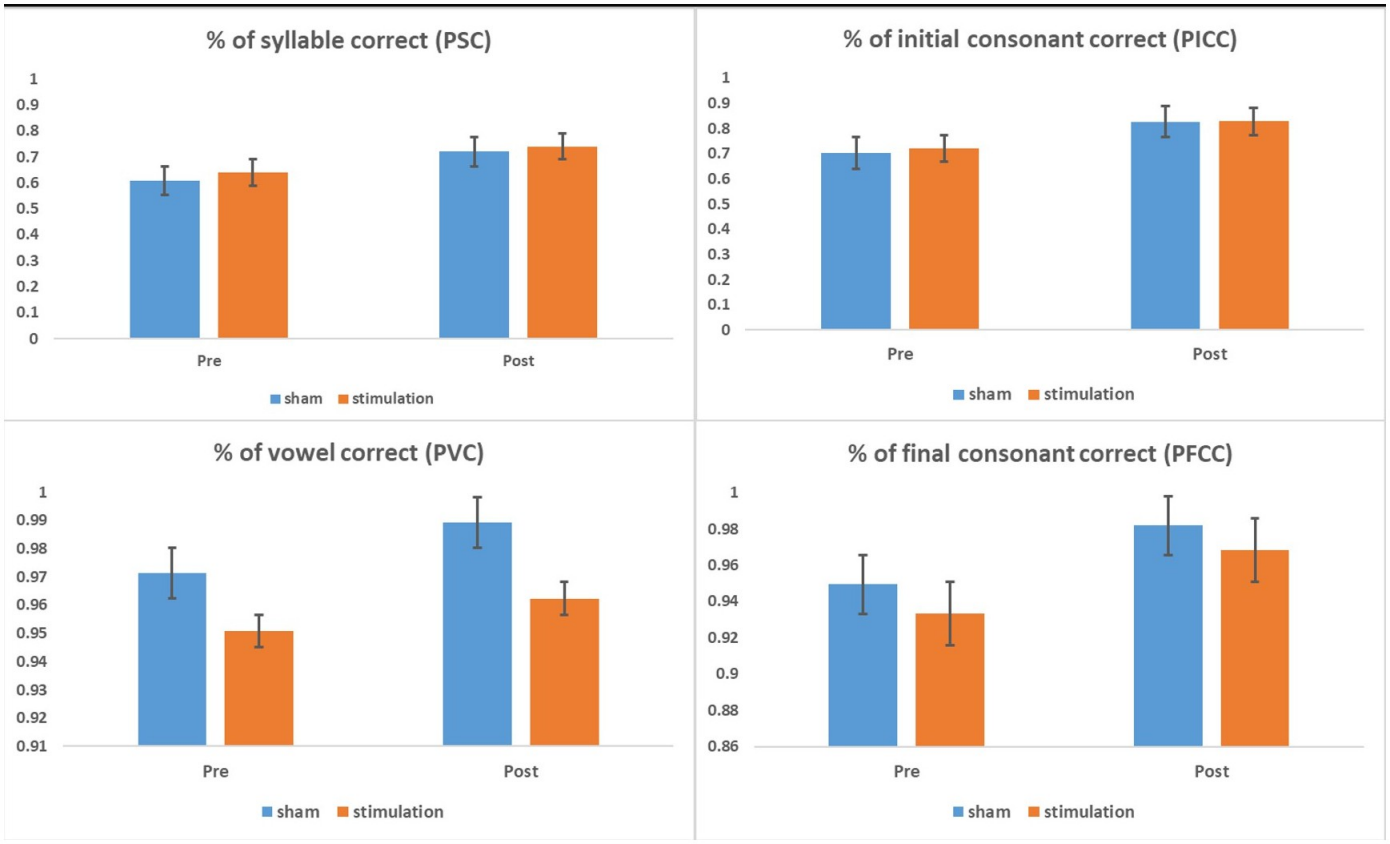
Mean (and standard error) perceptual ratings at the word level assessed using the Hong Kong Cantonese Articulation Test (HKCAT) for the stimulation and sham groups before and after treatment. Note: % = percentage.

Fig 4 displays the comparison of mean differences for the percentage of correctly produced syllable (PSC), initial consonant (PICC), vowel or diphthong (PVC), and final consonant (PFCC) among the stimulation and sham groups before and after treatment. Results showed significant main effects of time for PSC [*F*(1, 7) = 12.43, *p* = .01, *η*^*2*^ = .64] and PICC [*F*(1, 7) = 9.84, *p <* .05, *η*^*2*^ = .58] only. The percentage of correctly produced syllable and initial consonant were significantly higher post-treatment. However, no significant differences were found between the stimulation and sham groups. For the parameters PVC and PFCC, no significant main effects of time or group were found.

#### Passage reading

Table 3 presents the mean and standard deviation of the nineteen parameters measured in the perceptual evaluation of passage reading for the stimulation and sham groups before and after treatment. For perceptual analysis of passage reading, strong level of agreements among the 10 raters were reported in the intra-rater and inter-rater reliability (*K* = 0.86 and 0.85 respectively).

**Table 3:** Mean (and standard deviation) of the nineteen parameters measured in the perceptual evaluation of passage reading for the stimulation and sham groups before and after treatment.

Parameter	Stimulation group		Sham group	
	Pre	Post	Pre	Post
	Mean (*SD*)	Mean (*SD*)	Mean (*SD*)	Mean (*SD*)
Imprecise articulation	3.57 (1.45)	3.51 (1.94)	4.61 (2.32)	4.00 (1.90)
Irregular articulatory breakdown	3.28 (0.83)	3.94 (1.72)	3.17 (1.31)	3.36 (1.17)
Vowel distortion	3.18 (1.17)	4.08 (2.43)	3.06 (1.73)	3.75 (1.75)
Tone distortion	2.64 (1.09)	3.36 (1.64)	2.28 (1.29)	2.84 (1.32)
Hypernasality	1.58 (0.48)	2.22 (1.35)	1.66 (0.87)	2.19 (2.10)
Hyponasality	2.19 (1.96)	1.29 (0.34)	1.20 (0.32)	1.28 (0.34)
Short rushes of speech	1.75 (0.33)	1.38 (0.13)	3.06 (1.76)	2.37 (1.86)
Prolonged intervals	1.24 (0.18)	1.75 (0.33)	3.00 (1.73)	1.37 (0.13)
Prolonged phonemes	2.66 (1.86)	2.67 (1.72)	2.28 (0.63)	2.26 (0.58)
Repeated phonemes	1.72 (0.21)	2.02 (0.74)	1.71 (0.26)	1.89 (0.57)
Rough voice	2.37 (0.95)	2.39 (1.61)	2.28 (0.78)	2.41 (2.02)
Breathy voice	1.72 (0.42)	1.91 (1.10)	1.66 (0.58)	1.86 (1.22)
Strained-strangled voice	2.75 (1.29)	2.31 (1.32)	2.26 (0.95)	2.32 (1.93)
Monoloudness	3.19 (0.83)	2.83 (0.70)	2.84 (0.95)	3.20 (1.30)
Monopitch	3.15 (0.94)	2.88 (1.18)	3.20 (1.00)	3.40 (1.36)
Excess loudness variation	1.00 (0.00)	1.00 (0.06)	1.25 (0.25)	1.10 (0.12)
Excess pitch variation	3.50 (1.36)	1.00 (0.00)	1.25 (2.48)	1.06 (0.06)
Excess stress	2.42 (0.91)	2.42 (0.91)	2.25 (1.11)	2.14 (1.20)
Overall speech intelligibility	3.62 (1.20)	4.75 (2.14)	3.60 (1.50)	4.16 (1.80)

Results showed a significant main effect of time for short rushes of speech [*F*(1, 7) = 5.91, *p* < .05, *η*^*2*^ = .46], with a significant reduction observed at the post-treatment measure. However, no significant differences were found between the stimulation and sham groups. For the parameters monopitch [*F*(1, 7) = 6.41, *p* < .05, *η*^*2*^ = .48] and excess loudness variation [*F*(1, 7) = 8.95, *p* < .05, *η*^*2*^ = .56], significant interaction effects between stimulation condition and time were found. However, post-hoc analysis found that both the anodal and sham stimulations had no effect on monopitch and excess loudness variation over time. No significant main effects of time nor group was found in the remaining parameters measured.

#### Diadochokinetic rate

**Fig 5:**
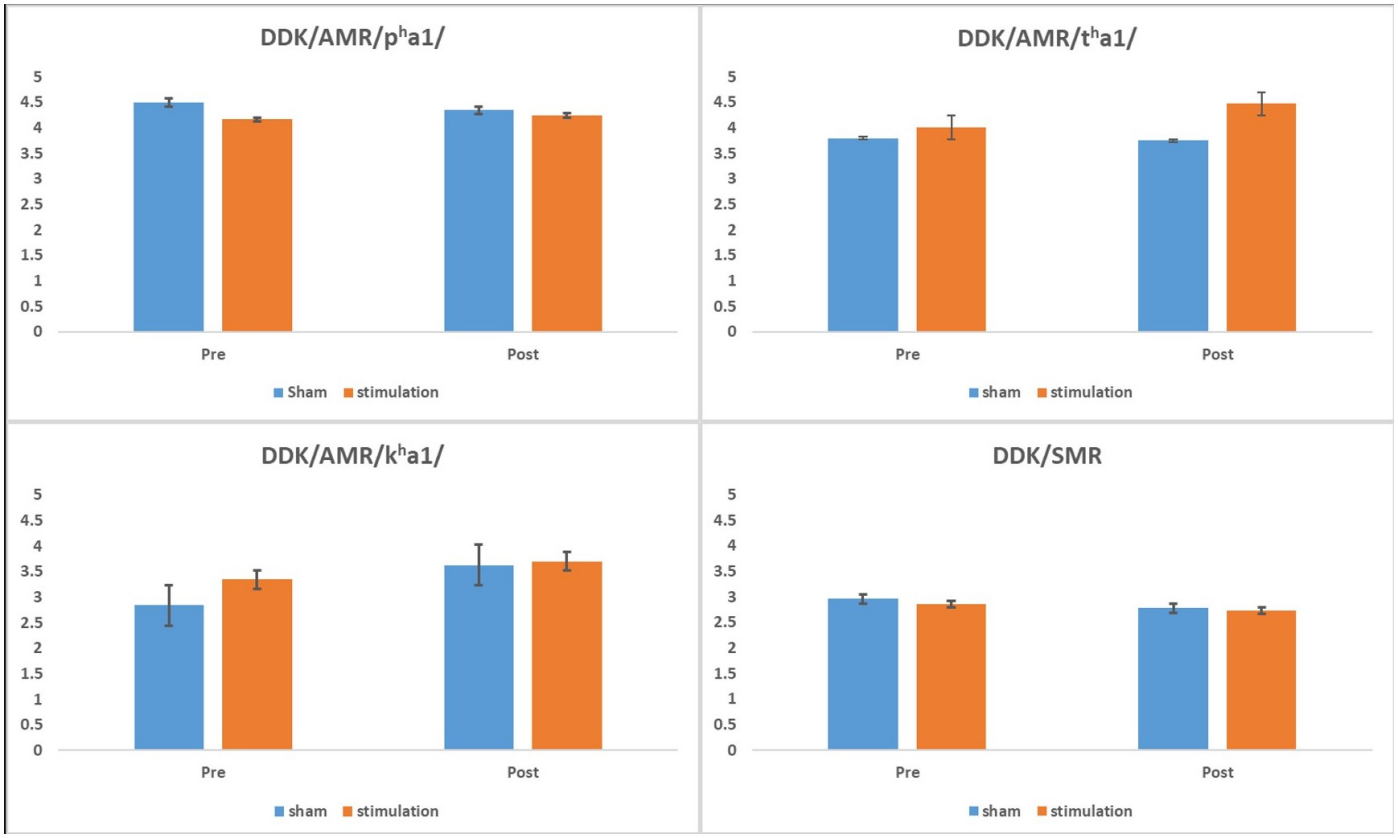
Mean (and standard error) DDK rates for the stimulation and sham groups measured before and after treatment.

Fig 5 displays the comparison of DDK rates mean differences between the stimulation and sham groups before and after treatment. Results showed a significant main effect of time for AMR-k^h^a1 [*F*(1, 7) = 6.41, *p* < .05, *η*^*2*^ = .48]. A significant increase in AMR-k^h^a1 repetition rate was observed at the post-treatment measure. However, there were no significant differences between the stimulation and sham groups. No significant main effects of time or group were found for AMR-p^h^a1, AMR-t^h^a1 and SMR-p^h^a1t^h^a1k^h^a1 repetitions rates.

### Acoustic analysis

Table 4 presents the mean and standard deviation of maximum phonation time and other voice-related acoustic measures for the stimulation and sham groups before and after treatment. Results showed a significant interaction effect between stimulation condition and time for shimmer [*F*(1, 7) = 10.32, *p* < .05, *η*^2^ = .60]. Post-hoc analysis showed that the stimulation group had a significant reduction in the shimmer values post-treatment [*F*(1, 4) = 7.84, *p* < .05, *η*^*2*^ = .66], while for the sham group, time had no effect [*F*(1, 3) = 3.27, *p* = .17, *η*^*2*^ = .52]. No significant main effects of time or group were found in all the other parameters measured.

**Table 4:** Mean (and standard deviation) maximum phonation time and other voice-related measures for the stimulation and sham groups before and after treatment.

Parameters	Stimulation group		Sham group		Cohen’s *d*
	Pre	Post	Pre	Post	
	Mean (*SD*)	Mean (*SD*)	Mean (*SD*)	Mean (*SD*)	
MPT (s)	12.65 (3.15)	13.83 (6.32)	10.02 (5.79)	11.25 (5.56)	
Jitter (local; %)	0.51 (0.45)	0.31^a^ (0.09)	0.38^a^ (0.20)	0.53 (0.36)	
Shimmer (local; %)	3.57 (1.86)	1.97^a^ (1.18)	3.65 (1.51)	4.68 (1.91)	1.76^
NHR ratio(dB)	0.05 (0.08)	0.03 (0.02)	0.04^a^ (0.07)	0.03 (0.02)	
HNR ratio (dB)	16.82 (5.82)	18.10 (3.57)	20.04 (1.04)	18.3 (2.02)	

Note: NHR = Noise-to-harmonics, HNR = Harmonics-to-noise.

^Effect size was calculated for parameter that showed significant interaction.

^a^Norms reported by Goy, Fernandes [56].

### Kinematic analysis

Tables 5 and 6 present the mean and standard deviation of kinematic parameters measured during DDK (rapid syllable repetition) and sentence production for the stimulation and sham groups before and after treatment. The kinematic parameters measured included duration, distance, maximum velocity, maximum acceleration and maximum deceleration in the approach and release phases.

**Table 5:** Comparison of kinematic parameters (DDK) between the stimulation and sham groups before and after treatment.

Kinematic parameters	Stimulation group				Sham group					
	Approach phase		Release phase		Approach phase		Release phase		App	Rel
	Pre	Post	Pre	Post	Pre	Post	Pre	Post		
	Mean	Mean	Mean	Mean	Mean	Mean	Mean	Mean	Cohen’s *d*	
	(*SD*)	(*SD*)	(*SD*)	(*SD*)	(*SD*)	(*SD*)	(*SD*)	(*SD*)		
**AMR/p** ^**h**^ **a1/**										
Duration (mm)	0.20	0.15	0.16	0.14	0.14	0.12	0.15	0.14		
	(0.09)	(0.07)	(0.05)	(0.05)	(0.03)	(0.03)	(0.04)	(0.04)		
Distance (ms)	14.75	14.29	14.93	14.03	11.50	9.56	11.01	9.35		
	(9.39)	(3.41)	(9.09)	(3.32)	(4.31)	(7.72)	(4.02)	(7.28)		
Maximum velocity (mm/s)	170.25	224.36	-199.90	-220.01	172.42	143.13	-151.27	-150.08		
	(93.4)	(126.56)	(175.76)	(68.83)	(84.37)	(126.54)	(57.03)	(128.55)		
Maximum acceleration (m/s^2^)	4850.37	7337.58	5514.95	7044.77	5693.63	4500.13	4897.74	4565.84		
	(2840.81)	(4941.34)	(3288.13)	(3751.77)	(2952.0)	(4000)	(2128.15)	(4355.34)		
Maximum deceleration (m/s^2^)	-4519.98	-7929.00	-5038.30	-5942.00	-5930.56	-4347.23	-5020.70	-4607.00		
	(2669.26)	(5617.00)	(2549.00)	(2013.00)	(3170.30)	(3333.74)	(1802.00)	(3561.00)		
**AMR/t** ^**h**^ **a1/**										
Duration (mm)	0.21	0.14	0.19	0.14	0.18	0.14	0.17	0.12		
	(0.07)	(0.05)	(0.06)	(0.05)	(0.05)	(0.07)	(0.03)	(0.04)		
Distance (ms)	11.02	10.67	10.95	10.05	9.83	7.81	9.79	7.60		
	(4.17)	(3.61)	(4.00)	(3.40)	(8.26)	(5.96)	(8.30)	(5.34)		
Maximum velocity (mm/s)	130.61	164.80	-157.42	-193.902	114.15	108.22	-133.85	-113.91		
	(47.34)	(48.37)	(69.54)	(47.48)	(88.35)	(79.50)	(113.54)	(81.30)		
Maximum acceleration (m/s^2^)	3179.78	5023.48	4238.84	6303.60	3214.99	3099.55	3466.49	3353.62		0.84^
	(1059.93)	(1594.26)	(1682.09)	(1641.90)	(1888.66)	(1899.06)	(2165.42)	(1970.25)		
Maximum deceleration (m/s^2^)	-5249.25	-7186.36	-5192.46	-6481.43	-4882.56	-3404.46	-4724.49	-3854.57		
	(2527.83)	(2646.60)	(2371.13)	(1265.90)	(3800.80)	(3228.00)	(3596.33)	(2225.17)		
**AMR/k** ^**h**^ **a1/**										
Duration (mm)	0.19	0.17	0.18	0.15	0.14	0.14	0.16	0.14		
	(0.07)	(0.08)	(0.05)	(0.06)	(0.02)	(0.06)	(0.02)	(0.04)		
Distance (ms)	11.94	11.70	11.30	10.90	2.10	4.90	2.05	4.92	0.69*	0.69*
	(6.70)	(4.80)	(6.80)	(4.19)	(0.72)	(3.10)	(0.55)	(3.10)		
Maximum velocity (mm/s)	115.00	146.50	-142.10	-149.10	38.07	67.24	-36.61	-78.70	0.89*	
	(33.4)	(42.35)	(76.24)	(25.19)	(7.40)	(40.00)	(9.04)	(48.92)		
Maximum acceleration (m/s^2^)	2967.13	4183.90	3395.50	4832.60	2560.17	2338.00	2474.21	2690.00	1.04*	0.78*
	(777.60)	(784.80)	(96.00)	(1271.20)	(360.40)	(1261.40)	(282.74)	(1567.00)		
Maximum deceleration (m/s^2^)	-3023.01	-4834.83	-3864.30	-4945.00	-2713.34	-2309.50	-2725.83	-2588.00		
	(757.00)	(1823.31)	(1898.00)	(472.60)	(340.13)	(1274.40)	(727.60)	(1103.30)		

Note: App = approach phase, Rel = release phase.

*Effect sizes were calculated for parameters that showed significant between-group differences.

^Effect size was calculated for parameter that showed significant interaction.

**Table 6:** Comparison of kinematic parameters (sentence) between the stimulation and sham groups before and after treatment.

Kinematic parameters	Anodal tDCS				Sham tDCS					
	Approach phase		Release phase		Approach phase		Release phase		App	Rel
	Pre	Post	Pre	Post	Pre	Post	Pre	Post		
	Mean	Mean	Mean	Mean	Mean	Mean	Mean	Mean	Cohen’s *d*	
	(*SD*)	(*SD*)	(*SD*)	(*SD*)	(*SD*)	(*SD*)	(*SD*)	(*SD*)		
**/p** ^**h**^ **a1/ in sentence**										
Duration (mm)	0.26	0.21	0.22	0.24	0.18	0.20	0.19	0.21	0.14^	
	(0.06)	(0.49)	(0.04)	(0.02)	(0.04)	(0.06)	(0.04)	(0.05)		
Distance (ms)	9.40	12.75	13.15	20.54	9.30	7.06	12.40	12.50	0.76^	
	(5.30)	(3.40)	(9.20)	(8.30)	(2.09)	(4.60)	(4.60)	(9.14)		
Maximum velocity (mm/s)	100.20	187.83	-156.31	-235.14	124.14	80.90	-156.12	-142.68	0.72^	
	(57.97)	(71.00)	(118.19)	(110.30)	(49.60)	(59.00)	(55.18)	(97.00)		
Maximum acceleration (m/s^2^)	4460.80	5000.00	3533.00	5346.00	4078.83	2528.34	4121.00	3240.00		
	(2968.00)	(3374.00)	(1990.00)	(2648.00)	(1408.00)	(1378.00)	(1075.00)	(2220.00)		
Maximum deceleration (m/s^2^)	-4979.33	-6832.84	-4081.01	-6421.95	-4191.31	-2751.93	-4572.19	-3780.62		0.46^
	(3686.00)	(3827.00)	(2063.00)	(2778.00)	(1990.00)	(1973.00)	(1412.00)	(2234.00)		
**/t** ^**h**^ **a1/ in sentence**										
Duration (mm)	0.20	0.20	0.24	0.83	0.19	0.17	0.19	0.60		
	(0.07)	(0.06)	(0.02)	(2.04)	(0.03)	(0.08)	(0.08)	(3.40)		
Distance (ms)	7.44	11.30	11.35	11.28	5.10	10.65	10.65	11.90		
	(2.90)	(3.10)	(3.10)	(6.27)	(4.50)	(7.30)	(7.51)	(8.50)		
Maximum velocity (mm/s)	69.90	97.70	-145.84	-169.24	71.80	56.50	-133.73	-156.76		
	(25.90)	(45.90)	(72.20)	(67.31)	(42.30)	(39.10)	(65.80)	(72.09)		
Maximum acceleration (m/s^2^)	2320.70	2748.85	3926.70	5165.57	1202.33	1748.41	4091.87	4705.60		
	(438.16)	(1123.00)	(2079.00)	(1915.60)	(2866.80)	(765.80)	(1742.36)	(905.00)		
Maximum deceleration (m/s^2^)	-3057.80	-3885.48	-4966.03	-6497.13	-2552.80	-2246.00	-3530.20	-4950.23		
	(1009.00)	(1834.60)	(2364.00)	(3029.70)	(1292.60)	(803.46)	(1511.00)	(2236.00)		
**/k** ^**h**^ **a1/ in sentence**										
Duration (mm)	0.16	0.18	0.32	0.29	0.17	0.17	0.21	0.15		0.74*
	(0.03)	(0.08)	(0.07)	(0.10)	(0.08)	(0.17)	(0.15)	(0.02)		
Distance (ms)	2.70	3.35	13.80	15.60	5.00	6.94	5.30	9.00		
	(1.36)	(2.04)	(9.40)	(8.10)	(4.00)	(7.70)	(3.70)	(3.10)		
Maximum velocity (mm/s)	32.30	47.60	-125.14	-165.83	115.30	68.20	-121.62	-148.65		
	(12.14)	(17.20)	(84.20)	(72.80)	(76.00)	(56.13)	(113.80)	(24.20)		
Maximum acceleration (m/s^2^)	1606.61	2021.30	2943.07	3520.00	12374.90	1639.49	7464.77	5510.00	0.21^	
	(433.00)	(152.00)	(1115.00)	(966.00)	(10372.00)	(2760.00)	(7747.00)	(1208.00)		
Maximum deceleration (m/s^2^)	-1417.57	-1818.19	-3256.75	-4080.00	-7635.22	-3052.24	-7917.42	-4848.23	0.66*	
	(452.00)	(564.00)	(1293.00)	(1300.00)	(3275.00)	(2281.10)	(8794.00)	(1012.00)		

Note: App = approach phase, Rel = release phase.

*Effect sizes were calculated for parameters that showed significant between-group differences.

^Effect sizes were calculated for parameter that showed significant interaction.

#### AMR-p^h^a1.

Results showed a significant interaction effect between stimulation condition and time for maximum deceleration in the approach phase of rapid /p^h^a1/ syllable repetition [*F*(1, 7) = 5.69, *p* < .05, *η*^*2*^ = .45]. Post-hoc analysis found that both the anodal and sham stimulations had no effect on maximum deceleration in the approach phase of rapid /p^h^a1/ syllable repetition over time. No significant main effects of time or group were found in other kinematic parameters measured during rapid /p^h^a1/ syllable repetition.

#### AMR-t^h^a1.

Results showed significant main effects of time for duration [*F*(1, 7) = 5.72, *p* < .05, *η*^*2*^ = .45] and maximum acceleration [*F*(1, 7) = 7.58, *p* < .05, *η*^*2*^ = .52] in the release phase of rapid /t^h^a1/ syllable repetition. Prolonged duration and increased maximum acceleration were observed in the post-treatment measures. However, no significant differences were found between the stimulation and sham groups. Significant interaction effects were found in maximum acceleration in the release phase of rapid /t^h^a1/ syllable repetition [*F*(1, 7) = 9.43, *p* < .05, *η*^*2*^ = .57] and maximum deceleration in the approach phase of rapid /t^h^a1/ syllable repetition [*F*(1, 7) = 6.23, *p* < .05, *η*^*2*^ = .47]. Post-hoc analysis found that the stimulation group had a significant increase in maximum acceleration in the approach phase of /t^h^a1/ syllable repetition post-treatment [*F*(1, 4) = 13.55, *p* < .05, *η*^*2*^ = .77] while no effect of time were observed in the sham group [*F*(1, 3) = 0.10, *p* = .77, *η*^*2*^ = .03]. Additionally, both the anodal and sham stimulations had no effect on maximum deceleration in the approach phase of rapid /t^h^a1/ syllable repetition over time. No significant main effects of time or group were found in other kinematic parameters measured during /t^h^a1/ syllable repetition.

#### AMR-k^h^a1.

Results showed that, in the approach phase of rapid /k^h^a1/ syllable repetition, the stimulation group had significantly longer distance [*F*(1, 7) = 23.20, *p* < .01, *η*^*2*^ = .77], increased maximum velocity [*F*(1, 7) = 50.33, *p* < .001, *η*^*2*^ = .88] and increased maximum acceleration [*F*(1, 7) = 9.72, *p* < .05, *η*^*2*^ = .58] than the sham group. In the release phase of rapid /k^h^a1/ syllable repetition, the stimulation group had significantly longer distance [*F*(1, 7) = 20.57, *p* < .01, *η*^*2*^ = .75] and increased maximum acceleration [*F*(1, 7) = 6.80, *p* < .05, *η*^*2*^ = .49] than the sham group. However, for maximum velocity [*F*(1, 7) = 17.52, *p* < .01, *η*^*2*^ = .72] in the release phase and maximum deceleration in both the approach [*F*(1, 7) = 6.52, *p* < .05, *η*^*2*^ = .48] and release [*F*(1, 7) = 11.95, *p* < .05, *η*^*2*^ = .63] phases of rapid /k^h^a1/ syllable repetition, the stimulation group had significantly reduced kinematic parameter values when compared with the sham group. No significant main effects of time or group were found in the remaining parameters measured.

#### Sentence-p^h^a1.

Significant interaction effects between stimulation condition and time were found for duration [*F*(1, 7) = 9.78, *p* < .05, *η*^*2*^ = .58], distance [*F*(1, 7) = 6.82, *p* < .05, *η*^*2*^ = .49], maximum velocity [*F*(1, 7) = 15.81, *p* < .01, *η*^*2*^ = .69], maximum acceleration [*F*(1, 7) = 5.82, *p* < .05, *η*^*2*^ = .45] and maximum deceleration [*F*(1, 7) = 6.87, *p* < .05, *η*^*2*^ = .50] in the approach phase of /p^h^a1/ syllable production in sentence, and for maximum deceleration [*F*(1, 7) = 10.46, *p* < .05, *η*^*2*^ = .60] in the release phase of /p^h^a1/ syllable production in sentence. Post-hoc analysis found that, in the post-treatment measures, the stimulation group had significantly shorter duration [*F*(1, 4) = 20.00, *p* < .05, *η*^*2*^ = .83], longer distance [*F*(1, 4) = 8.58, *p* < .05, *η*^*2*^ = .68] and increased maximum velocity [*F*(1, 4) = 40.04, *p* < .01, *η*^*2*^ = .91] in the approach phase /p^h^a1/ syllable production in sentence, and reduced maximum deceleration [*F*(1, 4) = 14.43, *p* < .05, *η*^*2*^ = .78] in the release phase of /p^h^a1/ syllable production in sentence. No significant main effects of time or group were found in all the other parameters measured.

#### Sentence-t^h^a1.

Results showed a significant main effect of time for maximum deceleration in the release phase of /t^h^a1/ syllable production in sentence [*F*(1, 7) = 8.15, *p* < .05, *η*^*2*^ = .54]. Significantly reduced maximum deceleration was observed in the post-treatment measure. However, no significant differences were found between the stimulation and sham groups. No significant main effects of time or group were found in all the other parameters measured.

#### Sentence-k^h^a1.

Results showed significant main effects of time for maximum acceleration [*F*(1, 7) = 6.88, *p* < .05, *η*^*2*^ = .50] and maximum deceleration [*F*(1, 7) = 20.94, *p* < .01, *η*^*2*^ = .75] in the approach phase of /k^h^a1/ syllable production in sentence. Reduced maximum acceleration and increased maximum deceleration were observed in the post-treatment measures. Significant main effects of group were found in maximum deceleration in the approach phase [*F*(1, 7) = 9.95, *p* < .05, *η*^*2*^ = .59] and in duration in the release phase [*F*(1, 7) = 5.70, *p* < .05, *η*^*2*^ = .45] of /k^h^a1/ syllable production in sentence. The stimulation group had higher maximum deceleration and prolonged duration than the sham group. Significant interaction effects between stimulation condition and time were found in maximum velocity [*F*(1, 7) = 7.36, *p* < .05, *η*^*2*^ = .51], maximum acceleration [*F*(1, 7) = 8.03, *p* < .05, *η*^*2*^ = .53] and maximum deceleration [*F*(1, 7) = 29.73, *p* = .001, *η*^*2*^ = .81] in the approach phase of /k^h^a1/ syllable production in sentence. Post-hoc analysis found that both the anodal and sham stimulations had no effect on maximum velocity over time. The stimulation group had increased maximum acceleration post-treatment [*F*(1, 4) = 8.17, *p* < .05, *η*^*2*^ = .67] while the sham group had increased maximum deceleration post treatment [*F*(1, 3) = 21.13, *p* < .05, *η*^*2*^ = .88]. No significant main effects of time or group were found in other kinematic parameters measured during /k^h^a1/ syllable production in sentence.

## Discussion

The current study investigated whether tDCS over the left inferior primary motor cortex (C5) would enhance dysarthria recovery in chronic stroke patients, specifically on speech intelligibility, speech-related physiological functions, and vocal function. Previous studies were limited and their outcome measures primarily focused on MPT and DDK rates. The inclusion of perceptual, acoustic and kinematic analyses would give a more comprehensive understanding of the potential effects of tDCS on speech and voice production. It was hypothesis that anodal stimulation would enhance speech intelligibility compared to sham stimulation.

The findings from perceptual analyses showed that both the stimulation and sham groups showed improved articulation post treatment, including more accurate initial consonant and syllable production at the word level, reduced short rushes of speech during passage reading and faster rate in AMR-k^h^a1. However, no group differences were documented. This implies that the improved articulatory control observed at the single word level, passage reading and rapid syllable repetition is likely to be contributed by speech therapy than anodal stimulation. Although improved initial consonant and syllable production were observed at the single word level, no corresponding articulatory improvement were observed at the passage reading. This may be due to task effects as higher demand of articulatory control is needed in passage reading. It is also important to note that participants who demonstrated reading difficulty were allowed to repeat after the examiner in truncated segments which are shorter than the natural parsing, the motor demand on articulatory control might have been alleviated. Therefore, it is possible that the speech intelligibility in passage reading was not reliably reflected. The findings on DDK tasks are different from those reported by You et al [21] in which their stimulation group showed significant improvement in rapid syllable /pa/ repetition when compared with the sham group.

For acoustic analysis, it was noted that anodal stimulation led to reduced shimmer value in sustained vowel /a/ phonation at the post treatment measure. Shimmer describes the amount of perturbation in amplitude, and it is a general indicator of the regularity of glottal vibration. Shimmer value has been found to moderately correlated with hoarseness [57]. The finding of a significant reduction in shimmer value implies an improved voice quality and potentially decreased severity in perceptual speech rating. On the contrary, perceptual ratings of roughness and breathiness in passage reading revealed no significant reduction. Such contradictory findings may be explained by the different task demand involved in sustained vowel and passage reading. The rapid alternating articulatory movement required in passage reading poses a higher motor demand on articulatory control than sustained vowel, which could have influenced perceptual judgment and masked the improvement in voice quality. A similar notion has been discussed by Gerratt, Kreiman [58]. Additionally, a lack of change in the maximum phonation time may implies that the main effect of tDCS on speech was not exerted on the respiratory-phonation subsystem.

Kinematic parameters were assessed by observing the movement of the lower lip, tongue tip and tongue dorsum during rapid syllable repetition and syllable production in sentence. For rapid syllable repetition, between group differences were mainly observed in rapid /k^h^a1/ syllable repetition (AMR-k^h^a1) which involved movement of the tongue dorsum. It was observed that, when compared to the sham group, the stimulation group had primarily increased distance, maximum velocity and maximum acceleration in the approach phase of AMR-k^h^a1, and increased distance and mixed performance in speed parameters (maximum velocity, maximum acceleration, and maximum deceleration) in the release phase of AMR-k^h^a1. Additionally, anodal stimulation also led to increased maximum acceleration in the release phase of AMR-t^h^a1 production at the post treatment measure. When examining lip and tongue kinematics during syllable production in sentence, it was found that anodal stimulation led to increased distance and maximum velocity but shorter duration in the approach phase of /p^h^a1/ syllable production in sentence at the post treatment measure. The anodal stimulation also led to increased maximum acceleration in the approach phase of /k^h^a1/ syllable production in sentence post treatment. Between group comparisons revealed that the stimulation group had increased maximum deceleration in the approach phase and prolonged duration in the release phase of /k^h^a1/ syllable production in sentence, when compared to the sham group. These findings imply that the positive effect of anodal tDCS was more apparent in the stimulation group when compared to the sham group, and its effects were observed in the movement of lower lip, tongue tip as well as tongue dorsum. It was also noted that kinematic analysis was more sensitive in detecting subtle changes in articulatory kinematics as compared with perceptual analyses. It is also important to note the results in kinematic analyses were not always observed or supported by perceptual and acoustic measures.

Although different types of speaking tasks, ranging from MPT, DDK, single word, sentence to passage reading were included in the current study, the analysis performed for each task was not uniform and limited the comparison of performance across tasks. Even though medium to large effect sizes were reported for some parameters measured, it is important to note that the different speaking tasks involved in the current study are not spontaneous speech production. The potential effects of tDCS in enhancing speech intelligibility at the spontaneous speech level is yet to be explored.

### Limitations and future investigation

The findings from this pilot study should be interpreted cautiously. The present study only included a small sample size of nine participants and the participants demonstrated different severity of dysarthria ranging from mild to severe. There was also a great variability of the time between the onset of dysarthria and the amount of speech therapy received by the participants. The heterogeneity may have contributed to the high standard deviations observed in some parameters measured and affected the comparability between the stimulation and sham groups. The participants’ speech performance was assessed by an experienced, qualitied speech therapist before commencement of the experiments for planning of individualized speech therapy. Therefore, despite the difference in dysarthria severity, the amount and duration of speech therapy received by each participant, each of them received tailor-made speech therapy targeted their dominant speech problems.

Previous studies have shown that recovery period following stroke expands from 6 months to several decades depending on the extent of brain damage. The faster recovery rate is only associated in the acute stroke than chronic stroke patients. Brain structural and functional recovery following stroke is multifactorial including but not limited to the location and size of the lesion, age and associated comorbidities [59]. In our study, all the participants included were right-handed, had a unilateral, left sided brain lesion and had a chronic stroke. None of them had an acute stroke that could be associated with spontaneous recovery.

The presence of mild aphasia in four participants may also influenced their speech performance because repetition after examiner were allowed when they had difficulties naming or reading the stimuli. In terms of the scope of kinematic investigation, the present study only investigated the kinematics of plosive production, rendering the results not generalizable to other Cantonese consonants to account for the overall improvement in perceptual speech intelligibility. In addition, due to the small sample size, the effects of anodal tDCS on severity and type of dysarthria were not examined. A larger sample is needed for between-group comparison to yield more conclusive results. Moreover, medical record (including stroke severity or disability) and imaging data on the exact brain lesion site was unavailable and limited the exploration of possible correlation between the cortical excitability of the primary motor cortex and neuroplasticity of the lesion site.

Lastly, there are some known issues and limitations of tDCS procedures: (1) A sham condition often involves much lower current flow in terms of duration or intensity as compared to an active stimulation condition. The commonly accepted idea that participants cannot distinguish between real and sham stimuli has been questioned [60]. Even if people cannot cognitively distinguish between the two conditions, other characteristics, such as arousal, may still vary. Therefore, it is crucial for experimental designs to include appropriate control conditions such as using an electrode montage that does not target the same region of interest, or by using a different stimulation frequency or phasic alignment [61]; (2) The present study applied conventional tDCS in which the current is applied through large electrodes and causes a diffuse electric field, decreasing current density and focality over the target area. Future studies are recommended to use high definition-tDCS montages with smaller electrodes sizes to reduce electric field variability and increase in focality [62]; (3) When considering spatial specificity, it’s important to think about how conventional tDCS might affect other brain regions close to the target brain area. Effects from the conventional tDCS are probably the result of stimulation at the target brain area or a mix of the target area and adjacent brain regions due to the big size of electrodes used in this procedure [61]; (4) There are various stimulation paradigms that differ in how reference electrodes are positioned. Few studies choose to position the cathode over a "reference" region and the anode over a known target area. Others employ an extracephalic reference electrode montage, which may prevent some of the negative consequences of tDCS, such as unwanted reversal effects under the reference electrode. This may be crucial in therapeutic contexts where a homogenous shift in cortical excitability is necessary.

## Conclusion

The present study supported the hypothesis that tDCS could enhance conventional speech therapy treatment outcomes in post-stroke dysarthric patients. Despite both the stimulation and sham groups had improved perceptual speech intelligibility at the word level, reduced short rushes of speech and increased rate of /kha1/ syllable repetition, the participants receiving anodal tDCS stimulation demonstrated significant improvement in shimmer value and articulatory kinematics during rapid syllable repetition and syllable production in sentence. More studies with larger sample sizes are needed to further explore the effects of tDCS in speech recovery post-stroke.

## Supporting information

S1 Table Stimuli used in kinematic measurement. ^1^ The underlined characters are the target DDK syllables embedded in a sentence in a linguistically appropriate context.
(DOCX)

S2 Table Raw data from perceptual analysis of the Hong Kong Cantonese Articulation Test. (XLSX)

S3 Table Raw data from perceptual analysis of passage reading. (XLSX)

S4 Table Raw data from perceptual analysis of diadochokinetic rate. (XLSX)

S5 Table Raw data from acoustic analysis. (XLSX)

S6 Table Raw data from kinematic analysis. (XLSX)

S1 File The original approved study protocol. (PDF)

S2 File The Cantonese oral passage *“North wind and the Sun”* for passage reading task. (DOCX)

S1 Checklist The CONSORT 2010 checklist. (PDF)
