# Supplementary material for: Transcranial direct current stimulation over the primary motor cortex improves speech production in post-stroke dysarthric speakers: A randomized pilot study
Source: PLoS One. 2022 Oct 13;17(10):e0275779. doi: 10.1371/journal.pone.0275779 (PMC9560523; doi:10.1371/journal.pone.0275779)
Supplement: S1 Table — 1 The underlined characters are the target DDK syllables embedded in a sentence in a linguistically appropriate context. (DOCX) [file pone.0275779.s001.docx]

Stimuli used in kinematic measurement

|  | Cantonese stimuli | English translation |
| --- | --- | --- |
| AMR task | 趴趴趴趴趴… | pa pa pa pa pa…  (lie on the stomach) |
|  | 他他他他他… | ta ta ta ta ta…  (he) |
|  | 卡卡卡卡卡… | ka ka ka ka ka…  ([train] car) |
| Syllable production in Sentence^1^ | 詩詩拋完波再趴喺到 | Si Si lies on her stomach after throwing the ball |
|  | 我懷疑他是貪心的人 | I suspect he is a greedy person |
|  | 喺第二卡車等 | Waiting in the second car (of a train) |

^1^ The underlined characters are the target DDK syllables embedded in a sentence in a linguistically appropriate context
