# Supplementary material for: Transcranial direct current stimulation over the primary motor cortex improves speech production in post-stroke dysarthric speakers: A randomized pilot study
Source: PLoS One. 2022 Oct 13;17(10):e0275779. doi: 10.1371/journal.pone.0275779 (PMC9560523; doi:10.1371/journal.pone.0275779)
Supplement: S1 File — (PDF) [file pone.0275779.s007.pdf]

Research Protocol

A randomized controlled trial study of the use of transcranial direct current stimulation (tDCS) in treating dysarthria post-stroke

Background of research

Dysarthria refers to neurological motor speech disorders resulting from disturbances in muscular control (e.g. paralysis, weakness or incoordination) over the speech mechanism [1]. Symptoms may be manifested in breathing, phonatory, resonatory, articulation and prosodic aspects of speech production [2]. Dysarthric speech may sound mumbled, choppy, soft, slurred, slowed but rushed, with abnormal pitch and rhythm [2]. As a result, dysarthria is associated with reduced speech intelligibility. Its impacts on oral communication can be chronic and debilitating and has the potential to significantly affect a patient's quality of life, which can lead to social isolation and depression. Stroke patients often demonstrate various types of motor speech problems. Previous studies have revealed that about 22-60% of stroke patients suffered a dysarthria [2, 3]. Treating dysarthria and improving their speech intelligibility therefore is an important part of post-stroke rehabilitation. Although research has indicated that the age-standardized mortality rate of stroke in Hong Kong is lower than the global rate and is declining [4], stroke is still a prominent cause of permanent disability in adults, affecting the person who may be disabled as well as their family and caregivers. With the rapidly ageing population in Hong Kong, it is expected that about one third of the population will be 60 years or older by 2050. Hence, stroke and stroke related disability would remain a significant disease burden in Hong Kong [5].

The commonly occurring features of dysarthria post-stroke including imprecision of articulation, slowed speaking rate, monotonous speech and voice disturbances [3]. Traditionally, speech therapy often involves strategies that focus on reducing the physiological impairment of a particular speech sub-system, for example, modifying the prosody and rate of speech to increase speech intelligibility, improving articulatory function for speech as well as articulation training [6, 7]. Some studies have used the Lee Silverman Voice Treatment, a treatment program reported to be effective in helping dysarthric individuals with Parkinson's disease to improve articulation and voice function in patients with dysarthria post-stroke [8, 9]. However, there is currently a paucity of rigorous scientific evidence supporting the efficacy of traditional treatment in the management of dysarthria post-stroke [3, 6].

In recent years, transcranial direct current stimulation (tDCS) has been widely used as a non-invasive brain stimulation technique to modulate cognitive functions and motor behaviour in healthy and brain-damaged individuals [10-15]. Compared with other brain stimulation techniques (e.g., transcranial magnetic stimulation), tDCS has the advantage of being noninvasive, portable and inexpensive. The procedure has also been found to be safe, with no evidence in neuronal damage checked with serum neuron-specific enolase [16, 17], MRI of edema in the related neurons [18], EEG waveforms [19], and thermal changes [20]. During tDCS intervention, a low amplitude direct current (typically between 0.5 and 2.0 mA) is transmitted through the scalp and skull to a specific cortical region via two electrodes (i.e., anode and cathode) placed on the scalp. The nature of the effect depends on the polarity of the current. Generally, an anodal (excitatory) tDCS increases neural excitability, whereas cathodal (inhibitory) tDCS decreases neural excitability [16]. The mechanism of tDCS is relatively well understood. Physiologically, the presence of the direct current (electrical

potential) in the region alters the polarization characteristics of the neurons. According to the literature, it is believed that the neurons near the anode (positive pole) are excited, with resting membrane potential shifting towards depolarization and an increased rate of spontaneous neuronal firing. On the contrary, neurons near the cathode (the negative pole) are inhibited, with resting membrane potential shifting towards hyperpolarization and a reduced rate of neuronal firing [21, 22]. As a result of the change in polarization behaviour of the neuronal cells, various functions can be altered. Research in healthy individuals showed that anodal tDCS enhances motor learning [23] and visuomotor performance [24] as well as improves working memory [25], verbal fluency [26] and vocal reaction times [27]. On the other hand, cathodal tDCS may reduce performance in working memory task [28] or may not have significant effect on a learning task [23].

Over the years, the application of tDCS in the management of psychiatric diseases [29] and neurological diseases, including stroke, has been increasing [15]. Research showed that tDCS has the potential to modulate motor recovery [11, 12, 30, 31] and language rehabilitation in aphasia post-stroke [32-35]. In the domain of speech production, recent reports have highlighted the beneficial effects of anodal tDCS in speech repetition tasks. In a preliminary study, Marangolo, Marinelli [36] applied 20 minutes of anodal tDCS over the left inferior frontal gyrus of three chronic aphasic patients while performing a speech repetition task. The tDCS stimulation, together with language therapy, was delivered for five consecutive days. Greater response accuracy was reported following the anodic stimulation. Similar positive effects on articulation were reported in another study with eight chronic patients who underwent bihemispheric stimulation over the left and right frontal regions together with concurrent speech therapy [37]. Significant recovery as indicated by better accuracy and speed in speech articulation were documented.

However, the application of tDCS in the management of dysarthria post-stroke has been limited. To the investigators' knowledge, only one study had investigated the effect of tDCS in the management of dysarthria post-stroke [38]. tDCS and conventional speech therapy were delivered to six acute stroke patients with dysarthria for a total of 10 sessions over a two-week period. Beneficial effects of anodal tDCS in treating dysarthria were reported. Despite the reported positive effects of tDCS, the observed improvement may have been contributed by spontaneous recovery. Moreover, the inclusion of a very small number of patients in the study also limited the generalization of the findings of the study. Therefore, more studies are needed to evaluate the efficacy and effectiveness of tDCS in the treatment of dysarthria post-stroke.

The current proposal is guided by evidence that anodal tDCS has the ability to modulate motor recovery and speech production in stroke [11, 38]. Since dysarthria is a disorder of speech movement resulting from disturbances in muscular control over speech mechanism [2], anodal tDCS to the motor area may potentially be used as an additional treatment in the management of dysarthria post-stroke. Research also showed that the electrophysiological effects of tDCS would outlast the duration of stimulation by up to 90 minutes after sessions of 1 mA polarization lasting for 9 - 13 min [23, 39, 40] and up to 2 months after 5-10 sessions of 1mA anodal stimulation lasting for 20 minutes [35, 36].

In an effort to confirm the ability of tDCS in the enhancement of speech motor performance, a pilot study was carried out by PI and Co-Is to investigate the short term effect of anodal tDCS. The study aimed at examining the possible effect in enhancing articulatory skills in performing tongue twister production task in healthy individuals who are native speakers of

Cantonese. Seven university students aged 18-25 years old with normal hearing participated in the study. All participants were native speakers of Cantonese who have no history of speech disorders, language disorders, brain surgery, seizure or stroke, and any implanted electrical or metallic device in the body.

Anodal tDCS was delivered to F5 region of the extended International 10-20 system for EEG electrode placement, which corresponds best to Broca's area [41]. A mild direct current of 2 mA was delivered to each participant by using a constant direct current stimulator (Chattanooga Ionto, Salty Lake, US) via saline-soaked sponge electrodes (50 x 70 mm) (EasyPads, Soterix Medical Inc., New York, USA) for 15 minutes [42]. For each participant, baseline data were obtained 30 minutes before the tDCS began. The participants were required to produce ten tongue twisters as fast and accurately as possible. During the recording the tongue twisters were presented in a random order using a computer. To avoid learning effect, no practice was provided prior to the experiment. Immediately after the stimulation, the participants were presented with the same tongue twisters and they have to produce them as fast and accurately as possible. Simultaneous to the tongue twister production by the participants, acoustic signals were recorded using a high-quality microphone (SM59A, Shure, USA) and a pre-amplification system (USB Pre, M-Audio, USA). A sampling frequency of 44 kHz and quantization rate of 16 bits/sample was used. Response accuracy and vocal reaction times in producing each tongue twister was collected for before, immediately after and 3 hours after the stimulation. Outcome measures included percent articulation accuracy (calculated by dividing the number of words correctly pronounced by the total number of words x 100%). In addition, acoustic analysis allowed calculation of perturbation measures such as jitter (perturbation in fundamental frequency), and shimmer (perturbation in amplitude). Average speech rate values (in WPM) were also obtained by dividing the number of words with the total time lapsed. To assess the reaction time, vocal reaction time values were also calculated from the onset of the presentation of visual stimulus to the offset of the participant's response.

The various measurements were obtained three times: 30 minutes before tDCS (pre), immediately after tDCS (post 1) and 3 hours after tDCS (post 2). While data are still being analyze statistically, preliminary findings in both articulation accuracy and vocal reaction time consistently indicate improved articulation through tDCS. In particular, nonparametric Friedman test revealed that the average vocal reaction time values obtained after stimulation were significantly shorter than before stimulation (pre = 10.405, post1 = 8.085, & post2 = 7.948) ($p < 0.01$), with no significant difference between post1 and post2 measurements. Although accuracy measure showed no significant difference ($p > 0.05$), the average accuracy values were steadily increased from 7.19 (pre) to 8.19 (post1) to 8.76 (post2). In sum, anodal tDCS over the left frontal region including Broca's area (F5) can enhance the accuracy and reaction time of tongue twister production in healthy individuals and the effect was maintained immediately after the stimulation.

The goal of the proposal project

The aim of the proposed study is to examine the short-term and long-term effects of tDCS on speech intelligibility and speech related physiological functions in chronic stroke patients who suffered a dysarthria. The ultimate goal of this project is to understand the mechanism of tDCS-induced effects, its application to the current models of motor speech rehabilitation post-stroke as well as to determine if tDCS can be used as an additional treatment approach targeting dysarthria post-stroke. It is anticipated that tDCS is effective in helping stroke patients with dysarthria in improving their speech production, thus their intelligibility and

eventually their quality of life after stroke. In addition, the proposed study will examine the change in speech motor function by using a comprehensive array of perceptual, acoustical, kinematic, and physiological measurements.

It is hypothesized that, given the proven ability of tDCS to increase the cortical excitability in individuals post-stroke, tDCS to the orofacial area of the primary motor area (SM1) of the affected hemisphere would result in increased speech intelligibility and speech related physiological functions.

Research plan and methodology

Participants

Participants will include 60 adults who are at least 6-month after their initial stroke and have suffered a dysarthria post-stroke. Based on the study by You, Chun [38] with an effect size of 1.08, priori power analysis showed that at least a total of 40 participants (20 participants in each group) are needed. Taking into account an attrition rate of around 25%, the minimum sample size for this study is 50 participants. The requirement of 6 months post stroke is to avoid possible spontaneous recovery effect. As sex does not appear to be a factor in the study, both male and female participants will be recruited. Participants will be recruited from Queen Mary Hospital and Tung Wah Hospital. Standard inclusion/ exclusion criteria for speech research and exclusion criteria for tDCS as will be adopted in the proposed study. Individuals with a personal or family history of epilepsy or seizures; a history of a neurological condition, speech disorders, oro-maxillo-facial surgery involving the tongue and/or lip, severe cognitive impairment, severe aphasia, in an unstable or serious medical condition including heart disease, metallic foreign body implant and/or any medications that lower neural thresholds (e.g. tricyclines, antidepressants, neuroleptic agents, etc.) will be excluded from the study. All participants will be screened using Cantonese mini-mental state examination (MMSE) and speech and language screening to exclude patients with cognitive impairment, aphasia and apraxia of speech.

Methods

Definition and classification of dysarthria

Each participant will be required to read a standard Chinese paragraph to generate a speech sample. Five experienced speech-language pathologists, who are blinded to the neurological condition and history of each participant will analyze the speech samples independently using a Hong Kong-based perceptual rating scales devised by Whitehill, Ma, and Lee [43]. The rating scale includes 21 speech dimensions covering eight categories, including pitch, loudness, voice quality, resonance, rate, articulation, tone, and general impression. The speech samples will be rated using a seven-point equal-appearing interval scale. During the process of rating the speech samples, the speech-language pathologists will determine the presence, type and severity of any associated dysarthria based on the Mayo Clinic model [2]. In cases of discrepancies, the speech-language pathologists will discuss among themselves and produce a single consensus rating to be used in all further analysis.

Transcranial direct current stimulation

High-definition tDCS will be applied in 10 daily sessions during a 2-week period, administered on Monday to Friday. The anodal stimulation will be delivered to the primary motor cortex (SM1) of the orofacial area. Previous studies have reported that up to 2 mA DC stimulation can be safely applied over the human cortex for up to 20 minutes [42]. As such, the current strength selected for this study will be 2 mA and the stimulation will last for 15 minutes. For the sham tDCS group, the same setting of tDCS electrodes will be applied on the scalp, but the stimulation will only last for 30 sec in order to cause similar sensation on

the scalp as the other group.

The conventional tDCS is performed using large electrode pads, most commonly between 25-35 cm^2, which stimulates relatively broad areas of cerebral cortex located between the anode and cathode pads. Therefore, focal stimulation of the target cortical region, not involving stimulation of neighbouring anatomical areas, is difficult to achieve with this conventional diffuse tDCS technique. However, increased focality would be desirable in applying tDCS on post-stroke patients. First, it may help achieve beneficial clinical effects with larger effect sizes. Second, it may also contribute to the understanding of the specific cortical regions implicated in therapeutic action, which is difficult to dissect with conventional diffuse tDCS. Finally, a more focal intervention may also be associated with increased safety. It could potentially reduce the likelihood of side effects due to decreased stimulation of adjacent regions, thus allowing for stimulation with increasing intensity or repletion to enhance efficacy.

Speech and voice therapy

For both anodal stimulation group and sham stimulation group, standard speech and voice training will be delivered to the participants. The treatment targets will be chosen based on the speech assessment results and will include drilling of speech production and voice rehabilitation. Each therapy session will be carried out simultaneously as the tDCS stimulation and will be within 30 minutes. Other than the designated period, the participants should have received no other speech and voice therapy.

Study design

The proposed project will be a randomized, placebo-controlled double-blinded control study. Following recruitment, the participants will be randomly assigned to one of two groups: Group 1 (n = 30) will receive anodal tDCS stimulation and intensive speech and voice therapy; Group 2 (n = 30) will receive sham tDCS stimulation and intensive speech and voice therapy. Both objective and subjective measurements on speech and speech-related physiological function will be administered four times in this study. The data collection will include perceptual and acoustic measurements, kinematic measurements, strength and endurance measurements and dysarthria-related quality of life measurements. The assessment will be conducted prior to tDCS baseline, immediately post tDCS, and post tDCS at 1-month and 3-month to assess the treatment outcome.

<u>Data processing and analysis</u>

Primary outcome measure

Perceptual and acoustic speech assessments

All participants will be required to produce a sustained vowel /a/, repeat some syllables (i.e., /pa/, /ta/, /ka/ and /pataka/), read a standard paragraph in Cantonese and have a two-minute conversation with the investigator. A professional grade microphone (SM58, Shure, USA) will be placed 10 cm in front of the participant's mouth to ensure the quality of recording from the interference of airbursts. Each task will be repeated five times for recording. The voice samples will be recorded by using a preamplification unit (MicroBook II, Motu, USA). The medial portion of the three trials will be selected for acoustic analysis using the PRAAT software, a signal analysis software suite.

For perceptual speech rating, five experienced speech-language pathologists blinded to the neurological condition and history of each participant will analyze the speech samples independently using the perceptual rating scales adopted by Whitehill, Ma [43]. The rating

scales include 21 speech dimensions covering eight categories, including pitch, loudness, voice quality, resonance, rate, articulation, tone, and general impression. The speech samples will be rated using a seven-point equal-appearing interval scale. During the process of rating the speech samples, the presence, type and severity of any associated dysarthria will be determined based on the Mayo Clinic model [2]. In cases of discrepancies, the speech-language pathologists will discuss among themselves and produce a single consensus rating to be used in all further analysis.

Acoustic analysis will be carried out using the PRAAT software. Acoustic measurements will include fundamental frequency (F0), variability of fundamental frequency (vF0), frequency perturbation (jitter %), intensity perturbation (shimmer %), relative average perturbation (RAP), and noise to harmonic ratio (NHR) to be obtained from both sustained vowel phonation and connected speech.

Secondary outcome measures

Kinematic Measurements

The lip and tongue function during speech production will be traced real time and objectively measured using the Enhanced Wave Speech Research System (Wave) (Northern Digital Inc., Canada). The Wave uses advanced 3D electromagnetic tracking technology to simultaneously track up to 16 sensors in five degrees of freedom (5 DOF). Movement is tracked in real time within a large 500 mm cubic measurement volume (electromagnetic field) with a sampling rate of 400 Hz. No line of sight is needed between the system and the subject to maintain accurate and reliable dimension and position tracking. The Wave is a non-invasive system and it meets the IEEE standards for electromagnetic radiation for head and torso tracking. The sensors are lightweight, ultra-small and disposable, can be safely attached to the subject using standard medical glue or adhesives and have little impact on articulation during speech production. All participants will be required to produce single-syllable real words of CV construction at high level tone embedded in a carrier phrase and repeat some syllables (i.e., /pa/, /ta/, /ka/ and /pataka/). The displacement, velocity, acceleration and duration of lips and tongue movement during speech production will be analysed.

Strength and endurance assessment

The strength and endurance of lip and tongue will be objectively measured using the Iowa Oral Performance Instrument (IOPI). Tongue strength will be assessed by asking the participant to "squeeze as hard as you can" with the anterior dorsum of the tongue against the IOPI tongue bulb placed against the hard palate. Lip strength will be assessed by asking the participant to squeeze their lips as hard as they can against the IOPI bulb placed between the lips. Tongue and lips endurance will be assessed by asking the participant to maintain 50% of maximal pressure (Pmax) as long as possible. Visual feedback will be given using the PicoScope 6 (Pico Technology, UK). The experimenter will provide spirited verbal encouragement throughout the trial. The trials will be timed with a stopwatch.

Quality of Life measurement:

The Chinese version of The Quality of Life of the Dysarthric Speaker (QOL-DyS) questionnaire [44] will be used to assess participants' subjective speech related quality of life. This self-reported questionnaire was adapted by the PI and Co-Is and is currently being validated. It includes 40 items covering four aspects of speech characteristics, functioning and well-being measurement including speech characteristics of the words, situational difficulty, compensatory strategies and perceived reaction of others.

Statistical analyses

Two-way repeated-measures Analyses of Variance (ANOVAs) will be applied for each dependent variable. Post-hoc multiple comparisons will be carried out when necessary.

1. Darley, F.L., A.E. Aronson, and J.R. Brown, *Differential diagnostic patterns of dysarthria.* Journal of Speech and Hearing Research, 1969. **12**: p. 246-269.
2. Duffy, J.R., *Motor speech disorders: Substrates, differential diagnosis, and management*. 3rd ed. 2013, St. Louis, MO: Elsevier Mosby.
3. Mackenzie, C., *Dysarthria in stroke: A narrative review of its description and the outcome of intervention.* International Journal of Speech-Language Pathology, 2011. **13**(2): p. 125-136.
4. Ng, P.W., *The stroke epidemic.* Hong Kong Medical Journal, 2007. **13**: p. 92-94.
5. Woo, J., et al., *Stroke incidence and mortality trends in Hong Kong: Implications for public health education efforts and health resource utilisation.* Hong Kong Medical Journal, 2014. **20**(Supplement 3): p. S24-29.
6. Yorkston, K.M., et al., *Evidence for effectiveness of treatment of loudness, rate, or prosody in dysarthria: A systematic review.* Journal of Medical Speech-Language Pathology, 2007. **15**(2): p. xi-xxxvi.
7. Yorkston, K.M., et al., *Managment of motor speech disorders in children and adults*. 2nd ed. 1999, Austin, Texas: Pro-Ed.
8. Wenke, R.J., P. Cornwell, and D.G. Theodoros, *Changes to articulation following LSVT® and traditional dysarthria therapy in non-progressive dysarthria.* International Journal of Speech-Language Pathology, 2010. **12**(3): p. 203-220.
9. Wenke, R.J., D. Theodoros, and P. Cornwell, *The short- and long-term effectiveness of the LSVT® for dysarthria following TBI and stroke.* Brain Injury, 2008. **22**(4): p. 339 - 352.
10. Flöel, A., *Non-invasive brain stimulation and language processing in the healthy brain.* Aphasiology, 2012. **26**(9): p. 1082-1102.
11. Adeyemo, B.O., et al., *Systematic review of parameters of stimulation, clinical trial design characteristics, and motor outcomes in non-invasive brain stimulation in stroke.* Frontiers in Psychiatry, 2012. **3**(88).
12. Kim, D.-Y., et al., *Effect of transcranial direct current stimulation on motor recovery in patients with subacute stroke.* American Journal of Physical Medicine & Rehabilitation, 2010. **89**(11): p. 879-886.
13. Sparing, R., et al., *Enhancing language performance with non-invasive brain stimulation—A transcranial direct current stimulation study in healthy humans.* Neuropsychologia, 2008. **46**(1): p. 261-268.
14. Nitsche, M.A. and W. Paulus, *Transcranial direct current stimulation - update 2011.* Restorative Neurology & Neuroscience, 2011. **29**(6): p. 463-492.
15. Schulz, R., C. Gerloff, and F.C. Hummel, *Non-invasive brain stimulation in neurological diseases.* Neuropharmacology, 2013. **64**(0): p. 579-587.
16. Nitsche, M.A. and W. Paulus, *Sustained excitability elevations induced by transcranial DC motor cortex stimulation in humans.* Neurology, 2001. **57**: p. 1899-1901.
17. Nitsche, M.A., et al., *Level of action of cathodal DC polarisation induced inhibition of the human motor cortex.* Clinical Neurophysiology, 2003. **114**(4): p. 600-604.
18. Nitsche, M.A., et al., *MRI study of human brain exposed to weak direct current*

stimulation of the frontal cortex. Clinical Neurophysiology, 2004. **115**(10): p. 2419-2423.

19. Iyer, M.B., et al., *Safety and cognitive effect of frontal DC brain polarization in healthy individuals.* Neurology, 2005. **64**: p. 872-875.
20. Stagg, C.J., et al., *Polarity-Sensitive Modulation of Cortical Neurotransmitters by Transcranial Stimulation.* The Journal of Neuroscience, 2009. **29**(16): p. 5202-5206.
21. Arul-Anandam, A.P., C. Loo, and P. Sachdev, *Transcranial direct current stimulation - what is the evidence for its efficacy and safety?* F1000 Medicine Reports, 2009. **1**: p. 58.
22. Bindman, L.J., O.C.J. Lippold, and J.W.T. Redfearn, *The action of brief polarizing currents on the cerebral cortex of the rat (1) during current flow and (2) in the production of long-lasting after-effects.* The Journal of Physiology, 1964. **172**(3): p. 369-382.
23. Nitsche, M.A., et al., *Facilitation of implicit motor learning by weak transcranial direct current stimulation of the primary motor cortex in the human.* Journal of Cognitive Neuroscience, 2003. **15**(4): p. 619-626.
24. Antal, A., et al., *Facilitation of visuo-motor learning by transcranial direct current stimulation of the motor and extrastriate visual areas in humans.* European Journal of Neuroscience, 2004. **19**(10): p. 2888-2892.
25. Fregni, F., et al., *Anodal transcranial direct current stimulation of prefrontal cortex enhances working memory.* Experimental Brain Research, 2005. **166**(1): p. 23-30.
26. Cattaneo, Z., A. Pisoni, and C. Papagno, *Transcranial direct current stimulation over Broca's region improves phonemic and semantic fluency in healthy individuals.* Neuroscience, 2011. **183**(0): p. 64-70.
27. Fiori, V., et al., *"If two witches would watch two watches, which witch would watch which watch?" tDCS over the left frontal region modulates tongue twister repetition in healthy subjects.* Neuroscience, 2014. **256**(0): p. 195-200.
28. Berryhill, M.E., et al., *A selective working memory impairment after transcranial direct current stimulation to the right parietal lobe.* Neuroscience Letters, 2010. **479**(3): p. 312-316.
29. Demirtas-Tatlidede, A., A.M. Vahabzadeh-Hagh, and A. Pascual-Leone, *Can noninvasive brain stimulation enhance cognition in neuropsychiatric disorders?* Neuropharmacology, 2013. **64**(0): p. 566-578.
30. Hummel, F.C. and L.G. Cohen, *Non-invasive brain stimulation: a new strategy to improve neurorehabilitation after stroke?* The Lancet Neurology, 2006. **5**(8): p. 708-712.
31. Boggio, P.S., et al., *Repeated sessions of noninvasive brain DC stimulation is associated with motor function improvement in stroke patients.* Restorative Neurology & Neuroscience, 2007. **25**(2): p. 123-129.
32. Holland, R. and J. Crinion, *Can tDCS enhance treatment of aphasia after stroke?* Aphasiology, 2012. **26**(9): p. 1169-1191.
33. Fiori, V., et al., *Transcranial Direct Current Stimulation Improves Word Retrieval in Healthy and Nonfluent Aphasic Subjects.* Journal of Cognitive Neuroscience, 2011. **23**(9): p. 2309-2323.
34. Medina, J., et al., *Finding the right words: Transcranial magnetic stimulation improves discourse productivity in non-fluent aphasia after stroke.* Aphasiology, 2012. **26**(9): p. 1153-1168.
35. Marangolo, P., et al., *tDCS over the left inferior frontal cortex improves speech production in aphasia.* Frontiers in Human Neuroscience, 2013. **7**.
36. Marangolo, P., et al., *Electrical stimulation over the left inferior frontal gyrus (IFG)*

determines long-term effects in the recovery of speech apraxia in three chronic aphasics. Behavioural Brain Research, 2011. **225**(2): p. 498-504.
37. Marangolo, P., et al., *Bihemispheric stimulation over left and right inferior frontal region enhances recovery from apraxia of speech in chronic aphasia.* European Journal of Neuroscience, 2013. **38**(9): p. 3370-3377.
38. You, D.S., et al., *The Effects of Transcranial Direct Current Stimulation on Dysarthria in Stroke Patients.* Journal of Korean Academy of Rehabilitation Medicine, 2010. **34**(1): p. 10-14.
39. Nitsche, M.A. and W. Paulus, *Excitability changes induced in the human motor cortex by weak transcranial direct current stimulation.* The Journal of Physiology, 2000. **527**(3): p. 633-639.
40. Zheng, X., D.C. Alsop, and G. Schlaug, *Effects of transcranial direct current stimulation (tDCS) on human regional cerebral blood flow.* NeuroImage, 2011. **58**(1): p. 26-33.
41. Naeser, M.A., et al., *Research with rTMS in the treatment of aphasia.* Restorative Neurology & Neuroscience, 2010. **28**(4): p. 511-529.
42. Nitsche, M.A., et al., *Safety criteria for transcranial direct current stimulation (tDCS) in humans.* Clinical Neurophysiology, 2003. **114**(11): p. 2220-2222.
43. Whitehill, T.L., J.K.Y. Ma, and A.S.Y. Lee, *Perceptual characteristics of Cantonese hypokinetic dysarthria.* Clinical Linguistics & Phonetics, 2003. **17**(4/5): p. 265-271.
44. Piacentini, V., et al., *Reliability and Validity of an Instrument to Measure Quality of Life in the Dysarthric Speaker.* Folia Phoniatrica et Logopaedica, 2011. **63**(6): p. 289-295.
