# Supplementary material for: Transcranial direct current stimulation over the primary motor cortex improves speech production in post-stroke dysarthric speakers: A randomized pilot study
Source: PLoS One. 2022 Oct 13;17(10):e0275779. doi: 10.1371/journal.pone.0275779 (PMC9560523; doi:10.1371/journal.pone.0275779)
Supplement: S2 File — (DOCX) [file pone.0275779.s008.docx]

The Cantonese oral passage *“North wind and the Sun”* for passage reading task

**北風和太陽**

有一日，北風同太陽喺度拗緊究竟邊個比較犀利。呢個時候，咁啱有個著住一件大褸嘅路人經過。於是，佢地決定邊個能夠令到嗰個人剝咗佢件褸既話，邊個就贏。

北風盡力咁吹，但係越吹得大力，路人反而將件褸摟得愈緊。北風唯有放棄。輪到太陽出馬既時候，太陽猛力咁曬，曬到路人流哂大汗，而且即刻將件褸剝咗落嚟。最後，北風唯有承認太陽比佢犀利。(149 words)
